# Uncoupling memory impairments from autism-associated behaviors in Chd2 deficient mice

**DOI:** 10.1038/s41380-026-03539-x

**Published:** 2026-03-23

**Authors:** Sang Ho Yoon, Robert F. Hunt

**Affiliations:** 1 Department of Anatomy & Neurobiology, University of California, Irvine, CA 92617 USA; 2 Epilepsy Research Center, University of California, Irvine, CA 92617 USA

**Keywords:** Neuroscience, Autism spectrum disorders

## Abstract

Mutations in the chromatin remodeler, *CHD2*, are strongly associated with moderate to severe intellectual disability, autism and epilepsy, but the direct contribution of *CHD2* mutations to clinical phenotypes is poorly understood. We report developmental and sex-specific behavioral changes in mice carrying a heterozygous mutation in *Chd2*. Notably, *Chd2* mutants display a range of abnormal behaviors including impairments in multiple forms of memory and social interaction. Memory impairments and memory-relevant transcriptional changes observed in *Chd2*^*+/−*^ mice are largely recapitulated in both sexes by conditional *Chd2*^*+/−*^ in adulthood. However, deficits in social behaviors and neuromodulatory system genes remain largely unaffected in conditional mutants. Reductions in interneuron density were identified throughout the brain of *Chd2*^*+/−*^ mice, and the GABA_A_ positive allosteric modulator, L-838,417, was effective in treating abnormal social behavior. Our results suggest a postdevelopmental role for *Chd2* in memory whereas neuropsychiatric conditions may be driven by more complex circuit mechanisms involving sexually dimorphic disruptions in brain development.

## Introduction

Genetic variation in the chromatin regulator, *CHD2*, is a leading cause of intellectual disability (ID), autism spectrum disorder (ASD) and developmental and epileptic encephalopathy (DEE) [1–12]. *CHD2* encodes an ATP-dependent chromatin remodeling factor that can modulate gene expression by altering chromatin structure [6]. Pathogenic variants are typically de novo loss-of-function mutations leading to haploinsufficiency or premature truncation [6, 7, 12]. This gives rise to a heterogeneous spectrum of neurologic symptoms, such as seizures, cognitive impairments and poor social communication, which typically begins in early childhood and becomes more prevalent in adult patients [10]. A major unresolved issue is the extent to which *CHD2* mutations contribute to *CHD2*-related pathologies, either directly through reduced *CHD2* function in mature neurons or indirectly through a disruption of neural development.

Evidence from mouse and human stem cell-based models indicate that *Chd2* is indispensable for the production of forebrain neurons [13–15]. *Chd2*^*+/−*^ mice produce fewer neurons in the developing forebrain, resulting from a reduction of the precursor pools of both excitatory and inhibitory neurons [13, 15]. Adult *Chd2*^*+/−*^ mice have a broad dysregulation of genes involved in synapse organization, loss of synaptic inhibition and an enhancement of neuronal excitability and glutamatergic neurotransmission [15]. Consistent with these previous findings and phenotypes observed clinically, in vivo studies have reported aberrant brain rhythms and memory impairment in *Chd2*^*+/−*^ mouse models [15, 16]. Recent genome scans of Labrador Retrievers eliminated from the US Transportation Security Administration (TSA) canine breeding program further implicates *Chd2* in performance-related learning and behavior issues [17]. However, given the large phenotypic variability seen in humans, a systematic analysis is needed to define which translationally relevant behavioral phenotypes can be reproduced in *Chd2*^*+/−*^ mice.

Here, we took advantage of a conditional *Chd2* mutant mouse line to investigate the influence of *Chd2* haploinsufficiency produced in development or adulthood, sex and their relationship with behavioral outcomes related to ID and ASD. By implementing a comprehensive preclinical phenotyping pipeline, we uncover behavioral impairments in *Chd2*^*+/−*^ mice relevant to *CHD2*-related brain disorders, including most forms of memory and social interaction. Memory impairments are reproduced by *Chd2*^*+/−*^ in adulthood, suggesting they may be particularly sensitive to therapeutic interventions aimed at enhancing *Chd2* function, but social impairments may require targeting underlying biological mechanisms.

## Materials and methods

### Gene expression

RPKM values were obtained from human brain in Allen Human Brain Atlas [18] and mouse brain in GEO database accession number GSE142208 [19] and processed, as previously described [19].

### Ethics approval

All procedures followed the guidelines of the University Laboratory Animal Resources at the University of California, Irvine and adhered to National Institutes of Health Guidelines for the Care and Use of Laboratory Animals.

### Animals

Experiments were performed on adult male and female mice maintained in standard housing conditions on a 12 h light/dark cycle with food and water provided *ad libitum*. *Chd2*-flox mice (i.e., Chd2^tm1c(EUCOMM)Hmgu^ mice) were crossed with C57BL/6 J mice (Jackson Laboratories cat. no. 000664) and maintained for >20 generations on this background before experimentation. Offspring were crossed to an ACTB-Cre line (Jackson Laboratories cat. no. 019099) to generate *Chd2*^*+/−*^ mice, CAGGCre-ER^*+/−*^ to generate *Chd2*^*+/cKO*^ mice (Jackson Laboratories cat. no. 004682), Ai14 tdTomato reporter (Jackson Laboratories cat. no. 007908) to confirm Cre-recombination and a hemizygous GAD67-GFP knockin line maintained on a CD-1 background [20] to identify inhibitory neurons. Littermates that were missing the floxed *Chd2* allele or Cre-ER were used as controls and received identical tamoxifen injections; wildtype C57BL/6 J littermates served as controls for *Chd2*^*+/−*^ mice.

### Experimental design

Experiments were performed on male and female littermates between P55 and P139. Whole litters were used for behavior experiments, with male and female mice being tested on different days, separately from each other. No data or animals were excluded from analysis. Control and *Chd2*^*+/cKO*^ mice were treated with 150 mg/kg Tamoxifen (Sigma-Aldrich, T5648) prepared in corn-oil (Sigma-Aldrich, C8267) and delivered by intraperitoneal injection for consecutive 5 days. Experiments were performed 4 weeks after administration. For immunostaining and qPCR experiments, animals were randomly allocated to experimental groups. For qPCR analysis of Chd2 expression, we performed a replication experiment using separate groups of animals; no other replication studies were performed.

### qPCR

Hippocampus or frontal cortex was dissected from female mice, total RNA extracted using a RNeasy Plus Mini Kit (Qiagen, 74134) and reverse-transcribed with the RevertAid First Strand cDNA Synthesis Kit (Thermo Scientific, K1622) according to the manufacturer instructions. The resulting cDNA was subjected to qPCR analysis with the Applied Biosystems Quantstudio 6 or 7 using PowerTrack™ SYBR Green Master Mix for qPCR (Applied Biosystems, A46109). Reactions were repeated in triplicates. Relative expression levels were calculated using the 2^−ΔΔCT^ method with Actb or Gapdh as an endogenous control gene. All primers used in this study are listed in Supplementary table 1.

### Immunostaining

Mice were transcardially perfused with 4% paraformaldehyde (PFA) and free-floating vibratome sections (50 μm) were processed using standard immunostaining procedures [15]. All antibodies have been previously used for immunostaining analysis in brain. Primary antibodies were: NEUN (mouse, Sigma-Aldrich, MAB377, 1:500), DsRed (rabbit, Takara, 632496, 1:500), GFP (chicken, Aves-Lab, GFP-1020, 1:1000). Secondary antibodies were: Alexa 488, Alexa 546 and Alexa 647 (Invitrogen, 1:500). Sections were then mounted on charged slides (Superfrost plus; Fisher Scientific) with Fluoromount-G that contained DAPI. Fluorescently labeled sections (50 μm) were imaged using a Leica DM6 microscope with a x10 objective and counted using ImageJ, as described previously [15]. Fluorescently labeled cells were counted in every sixth coronal section (that is, 300 μm apart). Four sections were analyzed per brain region per animal and the values averaged to obtain a mean cell density (cells / mm^2^). For prefrontal cortex (PFC), a region of interest encompassing the anterior cingulate area (ACA) and the dorsal portion of the prelimbic cortex (PrL), defined by its direct interface with the ventral surface of the corpus callosum, was delineated for cell counts. The basolateral nucleus of amygdala (BLA) was used for cell counts in amygdala. CA1 of dorsal hippocampus was quantified within a fixed-size square region of interest positioned at the center of the stratum pyramidale (SP) and encompassing all CA1 sublayers.

### Behavioral testing

All mice were individually habituated to handling for about 5 min on 5 consecutive days before testing. Handling took place in the holding room where the mice lived. Mice were then tested in two separate groups, with males and females tested on different days. Group 1 was evaluated in the following sequence: y-maze, marble burying test, open field test, object location memory, object recognition memory, three-chamber social interaction test, elevated plus maze, forced swim test and fear conditioning. Group 2 was evaluated in social avoidance after acute social defeat. The first behavior task was started when mice were P83 (i.e., 4 wks after tamoxifen), and the last task when mice were P125. For sociability following L-838,417 treatment, mice experiments were started when mice were P107. We added this information to the methods. Two or three cohorts of animals were used for each group. Each experiment was conducted separately 2 days to 1 week apart and only one behavioral test was conducted per day. All behavior assays were conducted between 12 pm and 7 pm during the light phase of the light/dark cycle (lights off at 8 pm; lights on at 8 am). Mouse identities were coded, and all behaviors were performed using a video tracking system and analyzed using ANY-maze software by investigators who were blind to the genotype and treatment of the animals.

#### Y-maze

The y-maze (Panlab, model no. LE847) consisted of three identical enclosed arms (30 L × 6 W × 15 H cm) set at an angle of 120° to each other, with visual cues located above and outside the maze, but not within it. The orientation of the maze and start arm both remained constant, but the other and novel arms were counterbalanced across animals. The test consisted of two trials separated by 90 min. In trial 1 (exposure), mice were first placed at the end of the start arm and allowed to explore the maze for 10 min with one of the arms closed. Mice were returned to their home cage located away from the test apparatus for 60 min. In trial 2 (test), mice were again placed in the start arm and allowed to explore all three arms for 5 min. The floor of the maze was cleaned with 70% EtOH (v/v) between trials. Behavior was videotaped and time spent in each arm was quantified by ANY-maze software. The number and sequence of arms entered were recorded at a later date by an investigator blind to animal treatment or arm identities. Percent alternation was calculated as the number of alternations (entries into three different arms consecutively) divided by the total possible alternations (i.e., the number of arms entries minus 2) and multiplied by 100.

#### Marble burying

20 marbles (16 mm, 5.5 g) were evenly placed in a mouse cage (30 L × 15 W × 13 H cm) containing unscented bedding (5 cm depth). Mice were placed in the cage and their activity was recorded for 30 min. Marbles were considered buried if more than two-thirds of their surface is buried underneath the bedding. Any instances where marbles were subsequently uncovered or re-buried by the mouse during the recording were not included in the final count.

#### Object location and recognition memory

These tasks consisted of three phases: habituation, exposure and testing according to a previous protocol [15]. On day 1, animals were habituated individually to the open field arena for 10 min under dim overhead lighting conditions (45 lux). For object location memory (OLM), the arena was a grey box (40 L × 40 W × 35 H cm) with a vertical marking strip on one wall. For object recognition memory (ORM), a white round arena (40 cm diameter) was used. For the exposure session (day 2), two identical objects were placed in the open field. Animals were allowed to explore each object for 10 min. The arena and objects were cleaned with 70% (v/v) EtOH (OLM) or 1% acetic acid (ORM) between trials. A retention test was performed 24 h after the exposure session (day 3). For OLM, one object was placed in a different location during the test phase. The objects used were Falcon 50 mL conical centrifuge tubes (Fisher, Cat no. 14-432-22) filled with beach sand. For ORM, one object was replaced with a different object in the same location. The objects used were a 3D printed triangle cube and a 75 mL glass flask. A mouse was scored as exploring an object when its head was oriented toward the object within a distance of 1 cm or when the nose was touching the object. The relative exploration time was recorded and expressed by a discrimination index (DI = [*t*_novel_ – *t*_familiar_]/[*t*_novel_ + *t*_familiar_] × 100) where *t* represents time. Mean exploration times were calculated and the discrimination indexes between treatment groups were compared. To diminish bias, animals from each treatment group were evaluated on the same day in the same arena, and the location of the novel object was counterbalanced across animals.

#### Fear conditioning

To measure contextual and cued fear memory, fear conditioning was modified from previously described protocol [21]. Mice were individually placed into context A of a fear conditioning chamber (Med Associates, model no. MED-VFC-OPTO-M) for 450 s, and a single shock (0.45 mA, duration 2 s) was delivered at 208 s, 298 s, and 388 s with 85 dB tone (duration 30 s). On day 2, contextual memory was tested by placing the mice back into the context A for 5 min. Context A was identical to the training conditioning, except that no shocks or tones were presented. The chamber consisted of a stainless steel grid floor, bright white light illumination in the chamber, 70% EtOH odor, lights dimmed to 20 lux in the test room and animals were transported in a transparent plastic container. On day 3, mice were placed into context B, which consisted of the same chamber as context A with some of the visual cues from context A intact (e.g., rectangular box, stainless steel grid floor) but other cues were derived from a distinct context B (i.e., dim light illumination in the chamber, 1% acetic acid odor, red lab tape under the grid floor, black plexiglass triangle insert overhead, darken test room and animals were transported in a cardboard box). The test mouse was placed in context B for 5 min followed by tone presentation to measure the auditory memory. Animals from each treatment group were evaluated on the same day in the same chamber. Immediately after each session, mice were placed in a transport cage, walked back to the holding room and placed in a holding cage until experiments on all of the animals from the home cage were performed. Behavioral performance was recorded by digital video camera (sample rate, 30 fps). Freezing responses and duration were quantified using Video Freeze software (Med Associates) with motion threshold set at 18 au and minimum freeze duration set to 30 frames (1 s).

#### Three-chamber social interaction

Animals were tested in a rectangular three-chambered box with Methacrylate floor and transparent walls (Panlab, model no. LE894), according to a published protocol [21]. Each chamber was 42 L × 20 W × 22 H cm. The assay consisted of three 10 min phases: a habituation, sociability and social novelty test phase. Mice were first placed into the center chamber and allowed to explore all three chambers for 10 min along with empty grid enclosures in each side chamber (Panlab, model no. LE894A; 8 × 18 cm, 3 mm bars spaced 7.4 mm apart). Then, 30 min later, an unfamiliar mouse (age- and sex-matched C57BL/6 J mice that had never been seen before) was enclosed in one of the grid enclosures and placed in a side chamber. The grid enclosures allowed nose contact between the bars, but prevented fighting, and were attached to the bottom of the assay with double sided tape. An unfamiliar object (3D printed rectangular cube) was placed in the other enclosure. Mice were placed into the center chamber and allowed to explore the entire social test box for 10 min. For the sociability phase, a familiar mouse used for sociability test was placed in a side chamber and a novel mouse (unfamiliar, age- and sex- matched C57BL/6) was placed in the opposite side. Trials were video recorded and interaction time was measured by an investigator blind to the experimental outcome of each animal. The amount of time the subject animal spent in proximity (5 cm) of each enclosure was measured as interaction time. Social index was calculated as the [(t_Stranger1_ - t_Object_) / (t_Stranger1_ + t_Object_)] for sociability and [(t_Stranger2_ - t_Stranger1_) / (t_Stranger1_ + t_Stranger2_)] for social novelty. The chambers were cleaned with 70% EtOH (v/v) between trials.To diminish bias, animals from each treatment group were evaluated on the same day in the same arena, and the location of the unfamiliar mouse and object were counterbalanced across animals.

#### Acute social defeat

This task was performed as previously described [22]. CD-1 mice were individually housed in their home cage for 1 week before testing. We pre-screened for aggression before encountering subject mice, and CD-1 mice that attacked an intruder mouse within 20 s for 2 consecutive days were included in this experiment. Subject mice were introduced into the home cage of aggressive CD-1 mice for 5 min and physically defeated. Non-defeat control mice are age- and sex- matched to experimental mice and had no previous exposure to CD-1 mice. Defeat sessions were videotaped, and the amount of time defeated was scored for each animal. Twenty-four hours after acute social defeat, mice were placed in a novel arena (42 L X 20 W X 22 H cm) containing a littermate of the CD-1 aggressor that subject mice had never seen before housed in a grid enclosure and allowed to explore the arena for 5 min. The arena was divided into half and the farthest part of the contained CD-1 mouse was defined as the “far zone”. The interaction zone encompassed a 1 inch area around the enclosure. Video recordings were taken during the test and time in interaction and far zones were measured using ANY-maze software.

#### Elevated plus maze

The elevated plus maze apparatus (Panlab, model no. LE842) was comprised of two open arms (6 W × 29.5 L × 1.8 H cm) and two enclosed arms (6 W × 29.5 L × 40 H cm), elevated 65 cm above the floor. Mice were placed in the center platform always facing the same open arm. Test duration was 10 min under standard dimmed lighting conditions (45 lux). All data were collected and analyzed automatically using ANY-maze software.

#### Forced swim test

The mouse forced swim test was conducted identical to the method described previously [21]. Mice were placed individually into a glass cylinder (height = 40 cm, diameter = 15 cm) containing 22 cm of water (22–23 °C) for 6 min. The total duration of immobility was recorded during the last 4 min of the 6 min testing period. A mouse was considered to be immobile when it floated in an upright position and made only minimal movements to keep its head above water. Trials were video recorded and scored offline by an investigator blind to the experimental outcome of each animal.

### Drug administration

L-838,417 (Tocris, Cat no. 3250) was dissolved in 0.1% DMSO (Sigma, Cat no. 472301) diluted in sterile Saline Solution (Teknova, Cat no. S5825). Drugs were administered by an intraperitoneal injection at a dose of 0.05 mgkg^−1^ 30 min before the behavioral tests, as previously described [23].

### Statistical analysis

All statistical analyses were performed with GraphPad Prism 8 software. Data were compared by two-tailed *t*-test, paired *t*-test or two-way ANOVA for multiple comparisons. A Tukey’s HSD *post hoc* test was performed when appropriate. Because *Chd2*^*+/−*^ and *Chd2*^*+/cKO*^ mice were evaluated separately from each other and have slightly different genetic backgrounds, statistical comparisons were also performed separately with each genotype having its own littermate control group. No data were excluded from analysis. Data are expressed as mean ± s.e.m., and significance was set at *P* < 0.05. All statistical analyses were summarized in Supplementary tables 2 (qPCR) and 3 (behavior).

## Results

### *Chd2* expression in mouse and human

**Fig. 1:**
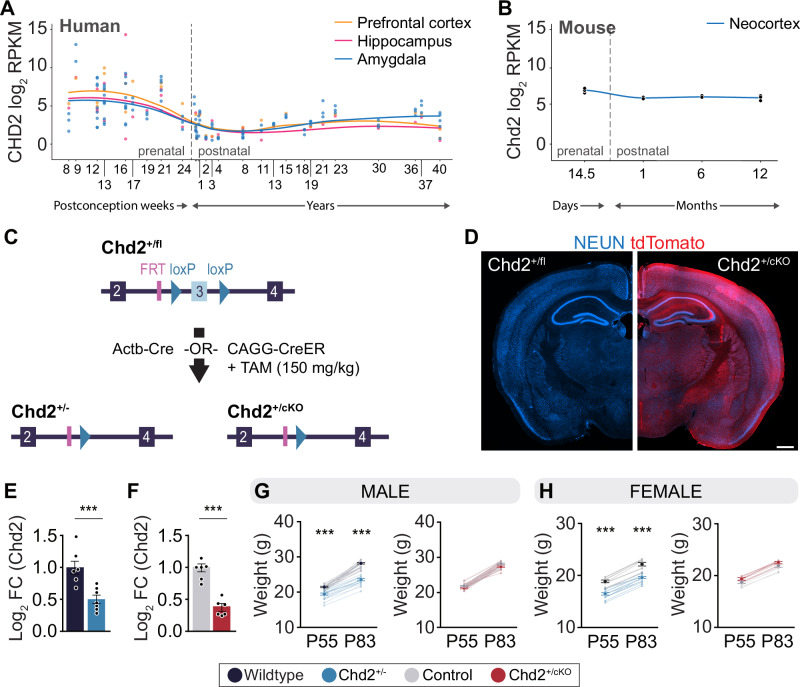
Generation of *Chd2* deficient mice. **A, B** *CHD2* gene expression in human brain (**A**) and mouse brain (**B**). **C** Schematic of the conditional allele for *Chd2*. Cre deletes the floxed exon 3 of *Chd2* to generate a frameshift mutation in development by crossing to Actb-Cre mice (i.e., *Chd2*^*+/−*^ mice) or adulthood by crossing to CAGG-CreER mice and administering 150 mg/kg tamoxifen for 5 days (i.e., *Chd2*^*+/cKO*^ mice). **D** Immunostaining for NEUN (blue) and tdTomato (red) in *Chd2*^*+/fl*^ and *Chd2*^*+/cKO*^ mice crossed to an Ai14 reporter line. Scale bar, 500 μm. **E, F** qRT-PCR validation of reduced *Chd2* expression in *Chd2*^*+/−*^ (**E**) and *Chd2*^*+/cKO*^ mice (**F**). ***P = 1.7E-04, wildtype vs *Chd2*^*+/−*^; ***P = 3.6E-05, control vs *Chd2*^*+/cKO*^; two-tailed t-test, *n* = 6-7 mice per genotype. **G, H** Body weight of male and female *Chd2*^*+/−*^ mice, but not *Chd2*^*+/cKO*^ mice, was reduced compared to wildtype littermates. Male wildtype vs *Chd2*^*+/−*^: ***P = 9.83E-09 at P55, *** *P* = 1.05E-15 at P83; female wildtype vs *Chd2*^*+/−*^: ***P = 3.27E-07 at P55, ***P = 5.62E-08 at P83; two-way rmANOVA with Tukey’s *post hoc* test, *n* = 8-23 mice per genotype. See Supplementary Table 2 for qPCR statistical analyses and Supplementary Table 3 for body weight analyses.

We used RNA sequencing data from human brain samples [18] to examine the regional expression of *CHD2*, including hippocampus, prefrontal cortex and amygdala (Fig. 1A). We detected robust *CHD2* gene expression from early-gestation (8 post-conception weeks, measured from the last menstrual period) to 40 year old adults. No major difference was noted between brain regions. In mice, the pattern of *Chd2* expression was qualitatively similar to human (Fig. 1B).

### Generation of *Chd2*^*+/−*^ and *Chd2*^*+/cKO*^ mice

To study the relationship between *Chd2* expression and behavior, we previously characterized mice with a near germline knockout in *Chd2* [15]. To establish a mouse line with heterozygous loss-of-function in adulthood (i.e., *Chd2*^*+/cKO*^ mice), we crossed transgenic mice containing loxP-flanked exon 3 of *Chd2* with a CAGG-Cre-ER^*+/−*^ line (Fig. 1C). Tamoxifen (150 mg/kg, 5 days) was administered at P55 for brain-wide activation of Cre-ER to drive reduced *Chd2* expression in *Chd2*^*+/cKO*^ mice. Littermates that were missing the floxed *Chd2* allele or Cre-ER were used as controls and received identical tamoxifen injections. In our study, we used heterozygous deletion to mimic the physiological expression of *CHD2* mutation seen clinically.

To confirm recombination, *Chd2*^*+/cKO*^ mice were crossed to an Ai14 reporter line. This revealed widespread tdTomato expression throughout the brain (Fig. 1D). As expected, *Chd2* expression was reduced by ~50% in hippocampus of both *Chd2*^*+/−*^ and *Chd2*^*+/cKO*^ mice (Fig. 1E, F). *Chd2*^*+/−*^ mice of both sexes had reduced body weight compared to wildtype littermates (Fig. 1G, H). In contrast, no weight differences were identified between control and *Chd2*^*+/cKO*^ mice on the first day of tamoxifen administration at P55 or 28 days later. Thus, decreases in body weight appear to be driven by *Chd2*^*+/−*^-related developmental delay.

### *Chd2*^*+/−*^ broadly disrupts memory

ID is one of the most prominent features of *CHD2*-related disorders [10], and *Chd2*^*+/−*^ mice perform poorly on object-based memory tasks [15]. However, it is unknown if these phenotypes can be reproduced by *Chd2*^*+/−*^ in adulthood, whether sex-differences exist or which forms of memory are most sensitive to changes in *Chd2* expression. Therefore, we first assessed behavioral performance in a range of memory behavior assays. Control and *Chd2*^*+/cKO*^ mice received tamoxifen at P55 and were tested starting 28 days later at P83. Age-matched wildtype and *Chd2*^*+/−*^ littermates were examined alongside these groups.

**Fig. 2:**
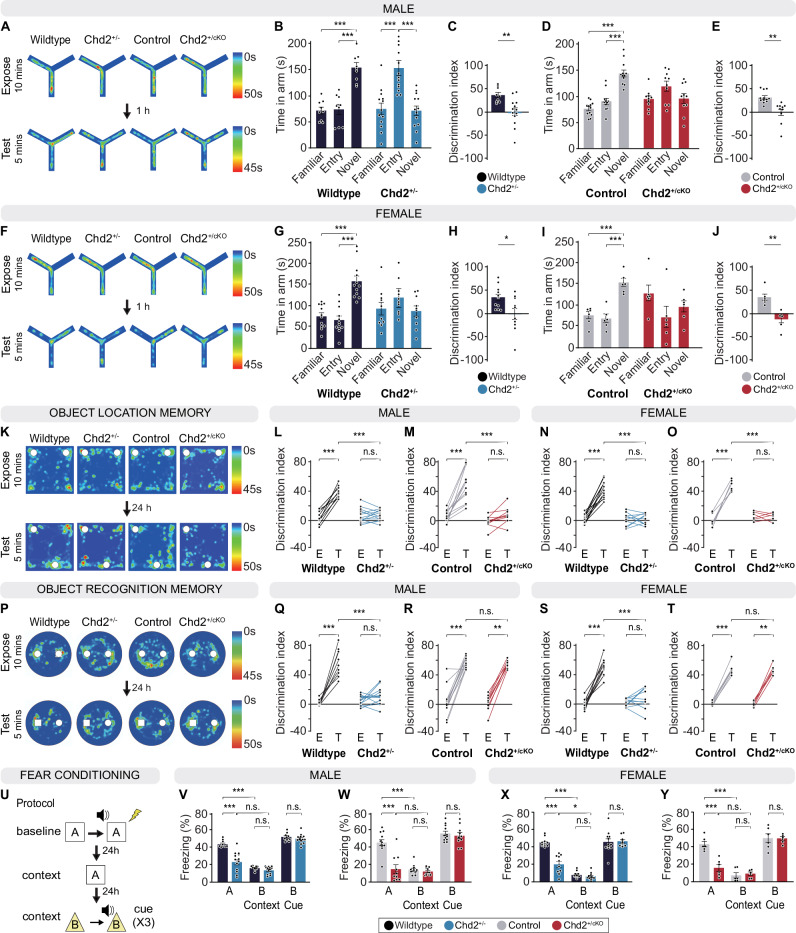
*Chd2* mutations disrupt multiple forms of memory. **A, F** Heatmaps showing the location of wildtype, *Chd2*^*+/−*^, control and *Chd2*^*+/cKO*^ mice during exposure and testing phases of the y-maze. **B, D, G, I** Quantification of time spent in each arm of the y-maze during the test phase. ***P < 0.001, two-way ANOVA with Tukey’s *post hoc* test, *n* = 6 – 13 mice per genotype. **C, E, H, J** Discrimination index for the novel versus familiar arm during the test phase. *P < 0.05, **P < 0.01, two-tailed t-test. **K** Heatmap showing the location of wildtype, *Chd2*^*+/−*^, control and *Chd2*^*+/cKO*^ male mice during exposure and testing phases of the object location memory (OLM) assay. **L-O** Discrimination index during exposure and testing phases of OLM for each individual mouse. ***P < 0.001, two-way rmANOVA with Tukey’s *post hoc* test. **P** Heatmap showing the location of male mice during exposure and testing phases of the object recognition memory (ORM) assay. **Q-T** Discrimination index during exposure and testing phases of ORM for each individual mouse. **P < 0.01, ***P < 0.001, two-way rmANOVA with Tukey’s *post hoc* test. **U** Schematic of the fear conditioning protocol. **V-Y** Quantification of freezing in contexts A and B for male (**V, W**) and female mice (**X, Y**). *P < 0.05, ***P < 0.001, two-way rmANOVA with Tukey’s *post hoc* test. See Supplementary Table 3 for statistical analyses.

To assess short-term memory in *Chd2*^*+/−*^ mice, we used a y-maze task in which mice were first exposed to the maze with one arm closed off. Then, one hour later, mice were re-introduced to the same maze and allowed to freely explore all three arms (Fig. 2A, F). During the test phase, male and female wildtype mice spent more time in the novel arm compared to familiar and entry arms (Fig. 2B, G). In contrast, *Chd2*^*+/−*^ mice did not show a preference for the novel arm and displayed a significantly lower discrimination index compared to wildtype controls (Fig. 2C, H). Male *Chd2*^*+/−*^ mice showed a preference for the entry arm whereas female mice showed no significant difference for time spent in each of the arms. This was a consistent result observed in across all cohorts of mice tested. The preference for the entry arm was observed in each cohort tested and was not due to immobility of male *Chd2*^*+/−*^ mice, because the overall distance traveled was not different among the groups (Supplementary Fig 1), or to decreased novelty seeking, as no differences were observed in total arm entries or alternations (Supplementary Fig 1). Similar to *Chd2*^*+/−*^ mice, male and female *Chd2*^*+/cKO*^ mice with conditional *Chd2* deletion in adulthood also showed reduced preference for the novel arm (Fig. 2D, E, I, J).

We next examined long-term spatial and recognition memory. In the object location task, wildtype mice exhibited increased exploration of the object that had been moved (Fig. 2K–O). In contrast, male and female *Chd2*^*+/−*^ mice explored both objects equally and displayed a significantly lower discrimination index compared to wildtype controls. *Chd2*^*+/−*^ mice showed similar deficits in the object recognition task (Fig. 2P–T). These results reproduce previously identified impairments of *Chd2*^*+/−*^ mice in object-based memory tasks [15]. However, unlike *Chd2*^*+/−*^ mice, male and female *Chd2*^*+/cKO*^ mice showed impairments only in object location memory (Fig. 2K–O), not object recognition memory (Fig. 2P–T). There was no difference in the time spent exploring the objects during the exposure or test phases of either task (Supplementary Fig 1), suggesting the poor performance of *Chd2* mutants was not due to disinterest in the objects.

**Fig. 3:**
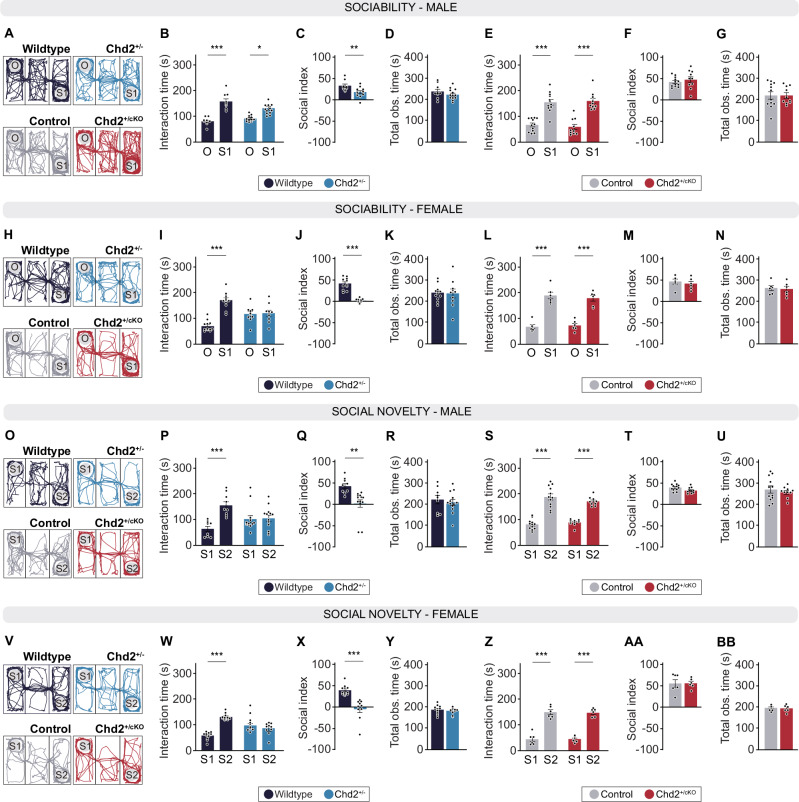
Social behavior deficits in *Chd2*^*+/−*^ mice are not reproduced in *Chd2*^*+/cKO*^ mice. **A, H** Tracking plot showing the location of wildtype, *Chd2*^*+/−*^, control and *Chd2*^*+/cKO*^ male (**A**) and female (**H**) mice during 3 chamber sociability testing. **B, E, I, L** Quantification of time spent interacting with object or stranger 1. *P < 0.05, ***P < 0.001, two-way ANOVA with Tukey’s *post hoc* test, *n* = 6 – 13 mice per genotype. **C, F, J, M** Social index during sociability test for male (**C**) and female (**G**). **P < 0.01, ***P < 0.001, two-tailed t-test. **D, G, K, N** Total time spent exploring the object and stranger mouse during sociability test. **O, V** Tracking plot showing the location of wildtype, *Chd2*^*+/−*^, control and *Chd2*^*+/cKO*^ male (**O**) and female (**V**) mice during 3 chamber social novelty testing. **P, S, W, Z** Quantification of time spent interacting with stranger 1 or 2. ***P < 0.001, two-way ANOVA with Tukey’s *post hoc* test. **Q, T, X, AA** Social index during sociability test. **P < 0.01, ***P < 0.001, two-tailed t-test. **R, U, Y, BB** Total time spent exploring the object and stranger mouse during sociability test. See Supplementary Table 3 for statistical analyses.

Finally, we used fear conditioning to evaluate cued and contextual memory. To do this, mice were placed into a fear conditioning chamber (context A), where they were exposed to a series of three tones (85db, 30 s duration, 90 s apart) followed 2 s later by electrical shocks (0.45 mA; 2 s duration) (Fig. 3U). Similar levels of freezing were observed among the four groups immediately following each foot shock (Supplementary Fig 1). Twenty-four hours later, mice were placed back into the same context A (with no shock). We found a significant reduction in freezing of *Chd2*^*+/−*^ and *Chd2*^*+/cKO*^ mice of both sexes, as compared to controls (Fig. 2V–Y), consistent with reduced fear memory. Freezing responses were comparable in male and female mice. The next day (24 h later), animals were placed into a similar context, B, in which the visual cues of context A were retained but other cues such as transport cage, odor, lighting and chamber shape were derived from a distinct context B. In this context, low levels of freezing were observed, and no significant difference was detected among the groups (Fig. 2V–Y). While control animals had significantly more freezing in context A than B, this difference was not observed in *Chd2*^*+/−*^ or *Chd2*^*+/cKO*^ mice of either sex. We then performed a cue test, where a series of three tones 60 s apart were presented immediately following context testing in B. Mice showed higher levels of freezing following the cue, and no differences were observed among the treatment groups (Fig. 2V–Y). Taken together, our findings demonstrate *Chd2*^*+/−*^ mice of both sexes have impairments in multiple forms of memory, and this is due, at least in part, to diminished *Chd2* function.

### *Chd2*-related social impairment is sex-dependent

Since ASD and other social behavioral issues are common in people with *CHD2*-related disorders [4, 5, 8–11], we next evaluated sociability and preference for social novelty. To do this, we employed a widely used three-chamber social interaction test. Following initial habituation to an empty arena, mice were exposed to an age- and sex-matched unfamiliar stranger mouse (S1) that was restricted to one of the lateral chambers. An inanimate object was placed in the opposite lateral chamber (Fig. 3A, H). Wildtype and control mice of both sexes spent significantly more time interacting with the stranger mouse (Fig. 3B, E, I, L). While male *Chd2*^*+/−*^ mice spent a significantly increased time with the stranger, females did not and mice of both sexes displayed a significantly lower social index compared to wildtype controls (Fig. 3C, J). The weaker preference for the mouse over the object indicates impairment in sociability that may be of greater magnitude in females versus males. In contrast, *Chd2*^*+/cKO*^ mice of both sexes were indistinguishable from controls; neither interaction time nor social index was different from controls. Total time spent interacting with the object and mouse was not different among the groups (Fig. 3D, G, K, N).

To further examine the preference for social novelty, each animal was retested in the three-chamber arena. In this test, the stranger mouse (S1) was restricted to one of the lateral chambers and a new unfamiliar stranger mouse (S2) was restricted in the opposite lateral chamber (Fig. 3O, V). Wildtype and control mice of both sexes spent significantly more time interacting with the novel stranger mouse (Fig. 3P, S, W, Z). *Chd2*^*+/−*^ mice did not show a strong preference for either mouse and displayed a significantly lower social index compared to wildtype controls (Fig. 3Q, X). *Chd2*^*+/cKO*^ mice of both sexes were again indistinguishable from controls (Fig. 3T, AA). Total time spent interacting with the stranger mice (S1 + S2) was not different among the groups (Fig. 3R, U, Y, BB). Taken together, these findings suggest sex-dependent impairments in social interactions that are likely driven by a disruption of brain development in *Chd2*^*+/−*^ mice rather than directly resulting from *Chd2* deficiency in the mature brain.

**Fig. 4:**
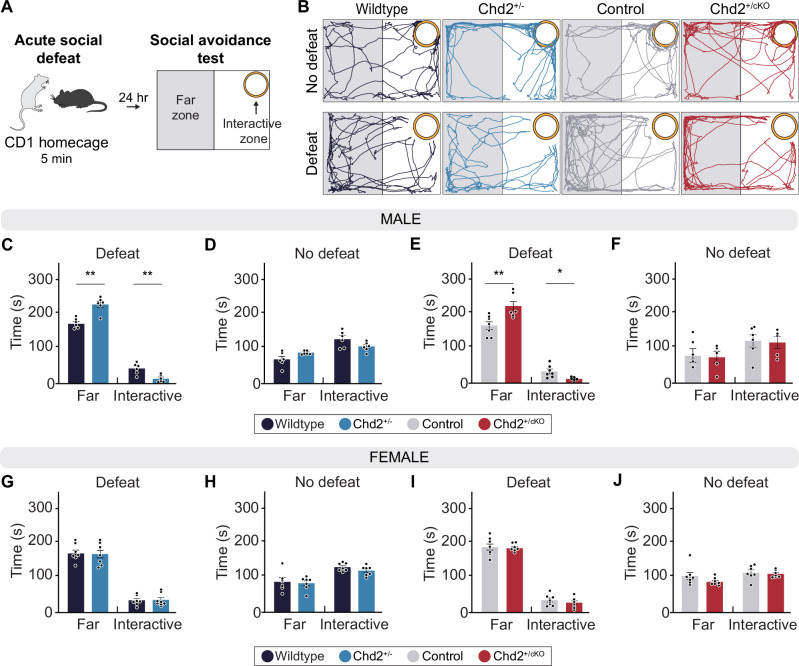
Male *Chd2* mutants exhibit increased avoidance behavior to an acute social defeat. **A** Schematic of social defeat protocol. **B** Tracking plots showing the location of wildtype, *Chd2*^*+/−*^, control and *Chd2*^*+/cKO*^ male mice exposed to CD1 mice that were aggressors (defeat) or not aggressors (no defeat). **C-F** Time spent by defeat (**C, E**) and no defeat (**D, F**) male mice in each zone. *P < 0.05, **P < 0.01, two-way ANOVA with Tukey’s *post hoc* test, *n* = 6 – 7 mice per genotype. **G-J** Time spent by defeat (**G, I**) and no defeat (**H, J**) female mice in each zone. *n* = 7 – 8 mice per genotype. See Supplementary Table 3 for statistical analyses.

Experiencing a physical attack from an aggressor leads to behavioral and physiological changes in the defeated mouse [22]. To investigate how *Chd2*^*+/−*^ mice respond to acute aggressive defeat, we subjected adult male and female mice to a 5-min bout of acute social defeat (aSD) in the home cage of a CD1 aggressor mouse of the same sex (Fig. 4A). Twenty-four hours after the aSD encounter, mice were placed into an arena that contained a novel, unfamiliar CD1 mouse and allowed to explore the arena for 5 min (Fig. 4B). When compared to controls, defeated male *Chd2*^*+/−*^ and *Chd2*^*+/cKO*^ mice spent significantly more time in the far zone of the arena and significantly less time in the interaction zone (Fig. 4C, E), suggesting greater avoidance behavior in male *Chd2* mutants. No difference among genotypes was detected in female mice (Fig. 4G–J) or mice of either sex that did not experience social defeat (Fig. 4D, F, H, J). There was no difference in time spent defeated among the groups (Supplementary Fig 2). Thus, in contrast to social novelty, we observed a sex-dependent response of *Chd2* mutants to aSD that was driven by diminished *Chd2* function.

### General activity and behaviors remain intact

Male and female *Chd2*^*+/−*^ mice did not display impairments in locomotor activity (open field test), anxiety (elevated plus maze), repetitive behavior (marble burying) or learned helplessness (forced swim test), and *Chd2*^*+/−*^ in adulthood did not affect these behaviors (Supplementary Fig 3).

### *Chd2*^*+/cKO*^ alters expression of synapse and memory-related genes

The poor performance of *Chd2*^*+/cKO*^ mice in memory tasks suggests these impairments result directly from reduced *Chd2* function in the mature brain. This could be explained by changes in gene expression related to synaptic plasticity or memory [24–26]. To test this, we first examined the expression of 40 genes involved in synapse organization or intellectual disability, including *Chd2*. Of these, 13 genes were previously found to be differentially expressed by RNA sequencing of adult *Chd2*^*+/−*^ mice [15] and 14 are putative interactors of *Chd2* [27].

**Fig. 5:**
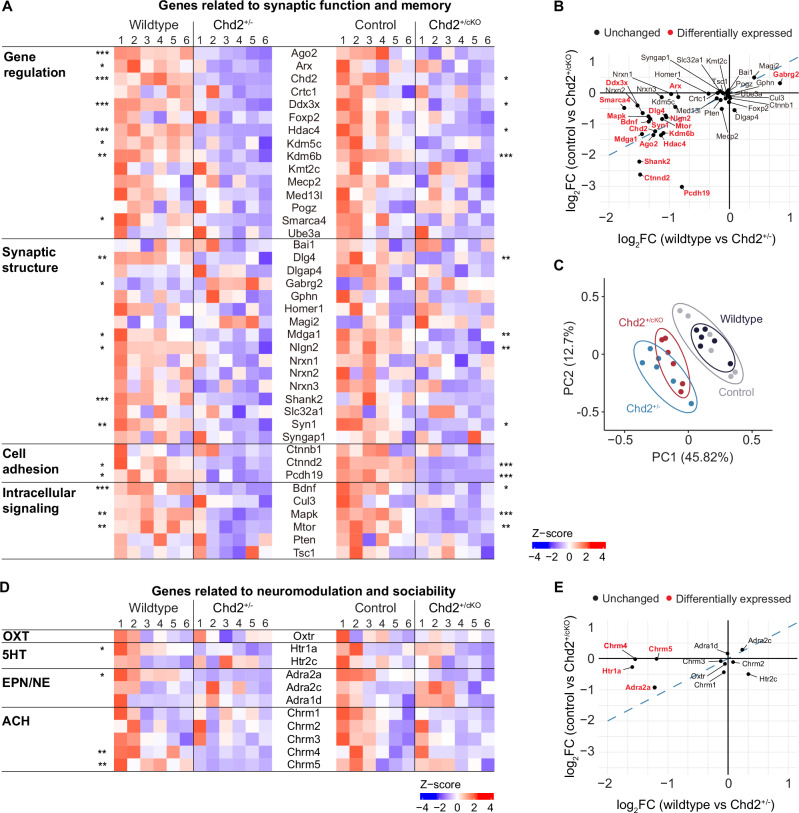
Transcriptional changes in *Chd2*^*+/−*^ mice are reproduced by *Chd2*^*+/cKO*^. **A** Heatmap of genes associated with synaptic function and memory. *P < 0.05, **P < 0.01, ***P < 0.001, two-way rmANOVA with Tukey’s *post hoc* test, *n* = 6 mice per genotype. **B** Scatterplot shows correlation between gene expression changes in *Chd2*^*+/−*^ and *Chd2*^*+/cKO*^ mice. **C** Visualization of qPCR data using principal component analysis (PCA). **D** Heatmap of genes associated with neuromodulation and social behavior. *P < 0.05, **P < 0.01, two-way rmANOVA with Tukey’s *post hoc* test, *n* = 6 mice per genotype. **E** Scatterplot shows correlation between gene expression changes in *Chd2*^*+/−*^ and *Chd2*^*+/cKO*^ mice. See Supplementary Table 2 for statistical analyses.

We performed qRT-PCR on hippocampus micro-dissected from 12 week old adult mice (4 weeks after tamoxifen administration for *Chd2*^*+/cKO*^ mice). We selected females for this experiment, because sex differences were not detected in any memory behavior. In *Chd2*^*+/−*^ mice, we observed broad differential expression of genes involved in cell adhesion (*Pcdh19*, *Ctnnd2*), intracellular signaling (*Bdnf*, *Mapk*, *Mtor*), synaptic function (e.g, *Dlg4, Mdga1*, *Shank2*) and gene regulation (e.g., *Ago2*, *Hdac4*, *Ube3a*) (Fig. 5A, Supplementary Fig 4A, B). This included changes in genes specific to glutamatergic synapses (*Dlg4*, *Shank2*), GABAergic synapses (*Gabrg2*, *Mgda1*, *Nlgn2*) and both synapse types (*Pcdh19*, *Syn1*). These results replicate prior expression changes detected in RNA sequencing of *Chd2*^*+/−*^ mice [15]. Most differentially expressed genes were reduced compared to wildtype littermates, consistent with the role of *Chd2* in promoting gene expression. Of the 19 genes with altered expression in *Chd2*^*+/−*^ mice, 13 (68%) of these were also decreased in *Chd2*^*+/cKO*^ mice (Fig. 5B). Principal Component Analysis (PCA) revealed data points from *Chd2*^*+/−*^ and *Chd2*^*+/cKO*^ mice clustered together and were distinct from the wildtype and control clusters (Fig. 5C). *Chd2* was also reduced by *Chd2*^*+/−*^ in adulthood, replicating our qPCR results in a separate cohort of mice.

Next, we examined the expression of 11 neuromodulatory system genes from frontal cortex that support social behavior. Of the four genes with decreased expression in *Chd2*^*+/−*^ mice (*Htr1a*, *Adra2a*, *Chrm4*, *Chrm5*), none were decreased in *Chd2*^*+/cKO*^ mice (Fig. 5D, E, Supplementary Fig 4C, D). These data suggest that *Chd2* deficiency in adulthood leads to a direct dysregulation of the molecular organization of synapses, and other genes associated with intellectual disability, independent from events occurring in early brain development. However, neuromodulatory systems remain largely unaffected by *Chd2*^*+/−*^ in adulthood.

### Fewer interneurons in *Chd2*^*+/−*^ but not *Chd2*^*+/cKO*^ mice

**Fig. 6:**
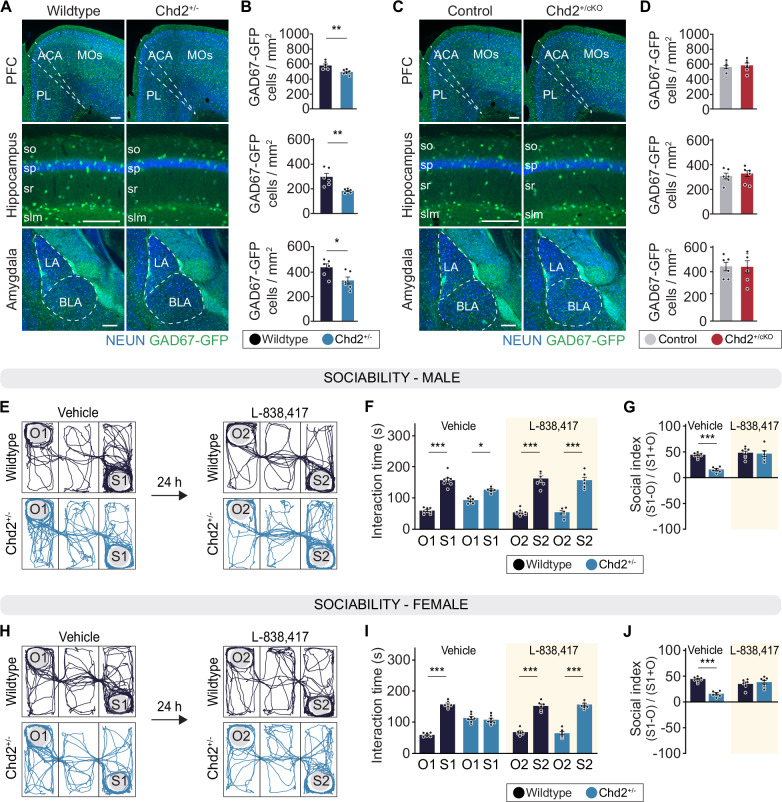
Treating *Chd2*^*+/−*^ mice with L-838,417 improves sociability. **A, C** Coronal sections of prefrontal cortex, CA1 region of hippocampus and amygdala immunostained for NEUN (blue) and GAD67-GFP (green). PFC, prefrontal cortex; ACA, anterior cingulate area; PL, Prelimbic; MOs, Secondary motor; so, stratum oriens; sp, stratum pyramidale; sr, stratum radiatum; slm, stratum lacunosum-moleculare. Scale bars, 200 μm. **B, D** Quantification of GAD67-GFP cell density in each brain region. PFC, **P = 0.004; Hippocampus, *P = 0.002; Amygdala, *P = 0.02; two-tailed t-test, *n* = 6 mice per genotype (3 male, 3 female). See Supplementary Fig 6 for breakdown of each sex. **E, H** Tracking plots showing the location of each group. O1, O2 and S1, S2 indicate different objects and stranger mice used for experiments. **F, I** Quantification of time spent interacting with object or stranger 1. *P < 0.05, ***P < 0.001, two-way ANOVA with Tukey’s *post hoc* test, *n* = 6 – 8 mice per group. **G, J** Social index during the sociability test. ***P < 0.001, two-way rmANOVA with Tukey’s *post hoc* test, *n* = 6 – 7 mice per group. See Supplementary Table 3 for statistical analyses.

Changes in inhibitory synapse genes could result from a loss of inhibitory neurons after tamoxifen administration, which are sensitive to reductions in *Chd2*; excitatory neuron loss is minimal or absent in *Chd2*^*+/−*^ mice [15]. Therefore, we quantified inhibitory neuron density in multiple brain regions involved in memory and social behavior. *Chd2*^*+/−*^ and *Chd2*^*+/cKO*^ mice were crossed to a GAD67-GFP reporter labeling nearly all GABAergic interneurons [20]. In *Chd2*^*+/−*^ mice, and in agreement with our previous findings [15], we observed ~20% reduction in GAD67+ neurons in hippocampus CA1, basolateral amygdala and prefrontal cortex (Fig. 6A, B). No overt sex differences were noted in any brain region (Supplementary Fig 5). In contrast, interneuron density was not significantly altered in *Chd2*^*+/cKO*^ mice (Fig. 6C, D). This suggests that behavioral phenotypes in *Chd2*^*+/cKO*^ mice are associated with changes in excitatory and inhibitory synapses but not reductions of interneuron numbers.

### Treatment with L-838,417 improves sociability

Decreases in GABAergic inhibition have been linked to social processing difficulties in neuropsychiatric conditions such as autism [23, 28]. Therefore, we asked whether treating *Chd2*^*+/−*^ mice with a GABA_A_ receptor modulator is sufficient to “rescue” social behaviors. To do this, we repeated the sociability assay with a second, independent cohort of *Chd2*^*+/−*^ mice and wildtype littermates treated with 0.1% DMSO in saline 30 min before testing. As expected, *Chd2*^*+/−*^ mice of both sexes had a significant decrease in sociability, as determined by a significantly lower social index compared to wildtype controls (Fig. 6E–J). The next day, the same mice were treated with a low dose of the α_2,3_-subunit-selective GABA_A_ positive allosteric modulator, L-838,417 (0.05 mgkg^-1^), and tested 30 min later using a different set of objects and stranger mice. Wildtype and *Chd2*^*+/−*^ mice treated with L-838,417 spent significantly more time interacting with the stranger mouse (Fig. 6E–J); L-838,417 treatment had no overt effect on wildtype animals, as expected [23]. There were no differences in the total time interacting with object and stranger or distance traveled during testing (Supplementary Fig 6). Thus, enhancing GABAergic transmission improves social deficits in *Chd2*^*+/−*^ mice.

## Discussion

The co-occurrence of ID and ASD is well established [29, 30], but molecular mechanisms underlying this comorbidity remain unclear. Here, we identified developmental and sex-specific behavioral outcomes linked to *Chd2* deficiency in mice. *CHD2* is one of the leading risk genes for ASD and ID [9]. Our results support a direct role of *Chd2* in multiple forms of memory. This is based on our observation that reduced *Chd2* expression in adulthood gives rise to transcriptional and memory impairments in the absence of neurodevelopmental changes. While abnormal social interactions were also seen in *Chd2*^*+/−*^ mice, these behaviors are sex-dependent and, except for social defeat in male mice, not reproduced by *Chd2*^*+/−*^ in adulthood. Our findings suggest a postdevelopmental role for *Chd2* in memory whereas neuropsychiatric conditions may require more extensive mechanisms involving sexually dimorphic disruptions in brain development.

*CHD2* deficiency has largely been considered a childhood epilepsy since its initial discovery in a population of DEE patients [3, 4], but clinical reports and natural history studies are increasingly documenting nonseizure outcomes in patients with or without epilepsy [10, 31, 32]. Adults with *CHD2*-related disorders present with considerable phenotypic variability, including ASD (79%), ID (71%), photosensitivity (64%), reduced mobility (57%), epilepsy (50%) and scoliosis (43%) [10]. Many, but not all, of these phenotypes can be modeled in *Chd2*^*+/−*^ mice. While spontaneous seizures have not yet been documented in any *Chd2*^*+/−*^ model system, we previously reported a dysregulation of neural oscillations and cross-cortical synchrony [15], with similarities to human epilepsy [33, 34] and ASD [35, 36]. Of note, many individuals with *CHD2* deficiency and ASD [37] or intellectual disability [32] also do not have epilepsy. Likewise, *CHD2* is expressed throughout the body, and patients often have musculoskeletal and autonomic dysfunction in addition to neurological symptoms [1–12]. How *Chd2* haploinsufficiency in other parts of the body influence brain function is currently unknown. While we previously reported mild lordokyphosis in *Chd2*^*+/−*^ mice [15], which reproduced a scoliosis-like phenotype first reported in a gene trap *Chd2*^*+/−*^ model [38], this phenotype was not apparent in *Chd2*^*+/cKO*^ mice. Together with our current results, our data provide a comprehensive preclinical behavioral phenotype of *Chd2* deficiency in mice.

A recent study reported only mild behavioral deficits in clasping and rotarod performance of *Chd2*^*+/−*^ mice maintained on a 129×1/SvJ genetic background [16, 39]; other relevant behaviors were not tested or not impaired in a way that mimics the clinical condition. Difficulty in observing behavioral phenotypes could be related to the 129 background mouse strain, which presents unique challenges for mouse behavior studies, the new mouse model employed or some other reason yet to be identified. For example, 129 strains generally perform poorly on locomotor, memory and autism-relevant behavior tasks versus other strains [40–42], possibly related to deletion of *Disc1* in all 129 strains [43]. Moderate callosal agenesis in this strain [40] could further have important implications for interpretation of EEG data, especially interhemispheric coherence.

Compared to wildtype littermates, male and female *Chd2* mutants displayed substantial reductions in short-term, spatial, recognition and contextual memory. Our finding that memory phenotypes – and reduced expression of genes involved in synaptic transmission – are reproduced by *Chd2*^*+/−*^ in adulthood suggests that *Chd2* is required to maintain full neurological function once development is complete. These data also suggest memory impairments might be treatable by elevating *Chd2* expression in the mature brain. The only memory behavior that was not reproduced by *Chd2*^*+/−*^ in adulthood was ORM, suggesting *Chd2*^*+/−*^ alone is insufficient for disrupting this task. ORM requires the coordinated interaction of many brain regions, including insular, perihinal, prefrontal cortex and hippocampus, and it is influenced by neuromodulatory activities [44]. While brain-wide recombination was achieved after tamoxifen administration, inhibitory interneurons and many neuromodulatory systems remain intact.

A growing body of evidence shows that restoring the expression of some ASD risk genes in adult mice can alleviate memory deficits [45–47]. Such a strategy was recently developed using an antisense oligonucleotide (ASO) targeting the long noncoding RNA, *Chaserr*, to increase *Chd2* expression in postnatal mice [39, 48], but effects on synaptic transmission or memory have not yet been demonstrated. The possibility of correcting intellectual disabilities in *CHD2*-related disorders is further supported by a prior study demonstrating an object-based spatial memory deficit could be corrected by transplanting inhibitory interneurons into hippocampus of *Chd2*^*+/−*^ mice [15]. Whether it would be more advantageous to restore *Chd2* function or manipulate disease mechanisms related to glutamatergic or GABAergic circuits for therapy is an open discussion. A detailed and direct analysis of how changes in *Chd2* expression affect the molecular architecture of individual synapses should clarify synaptic mechanisms driving *Chd2*-related phenotypes.

While ASD and social interaction disorders are well documented in *CHD2*^*+/−*^ patients [4, 5, 8–11], it is not yet known if sexual dimorphism of social behaviors also exists. It seems unlikely that the social behavioral outcomes identified in our experiments would be amenable to therapies aimed at enhancing *Chd2* expression alone. Unlike memory phenotypes, most social deficits could not be reproduced by conditional *Chd2*^*+/−*^ in adulthood. This suggests that social phenotypes may emerge from abnormal brain development rather than reduced *Chd2* expression alone. However, our data suggest targeting an underlying biological mechanism, such as a reduction in GABAergic inhibition, could be a promising therapeutic strategy for this disorder. Sex differences in social behaviors are influenced by a complex interaction of hormonal, genetic, epigenetic and environmental influences during brain development, which shapes the underlying neural circuitry [49–51]. Sexually dimorphic social phenotypes have been reported in other neurodevelopmental disorders [52–54]. While the neural mechanisms underlying sex differences in *Chd2*^*+/−*^-related social deficits are unknown, *Chd2* directly interacts with sex-specific genes associated with neurodevelopmental disorders, such as *Ddx3x* [27], which mediates sexual differentiation during brain development [55]. *Ddx3x* is differentially expressed in RNA sequencing of *Chd2*^*+/−*^ mice [15], qPCR analysis of *Chd2*^*+/−*^ and *Chd2*^*+/cKO*^ mice (Fig. 6), and as an X-linked gene, it would be expected to affect males more severely than females. Careful phenotyping of patients will better define sex-specific phenotypes of this disorder in human.

Our work adds to the growing body of evidence that *Chd2* mutations broadly disrupt behavior in multiple species, including mice, dogs and human. We further provide a translational framework that can be used for testing in vivo the effect of targeted treatments for *CHD2*^*+/−*^ that may be relevant to other chromatin-related disorders. Future studies will be critical to understand how molecular, cellular and synaptic alterations lead to *Chd2*-related phenotypes and can be targeted for therapy.

## Supplementary information

Supplementary Information

## Data Availability

All data are included in the manuscript.
