# Supplementary material for: Uncoupling memory impairments from autism-associated behaviors in Chd2 deficient mice
Source: Mol Psychiatry. 2026 Mar 23;31(8):4270–81. doi: 10.1038/s41380-026-03539-x (PMC13364646; doi:10.1038/s41380-026-03539-x)
Supplement: Supplementary file 1 — Supplementary Information [file 41380_2026_3539_MOESM1_ESM.docx]

**
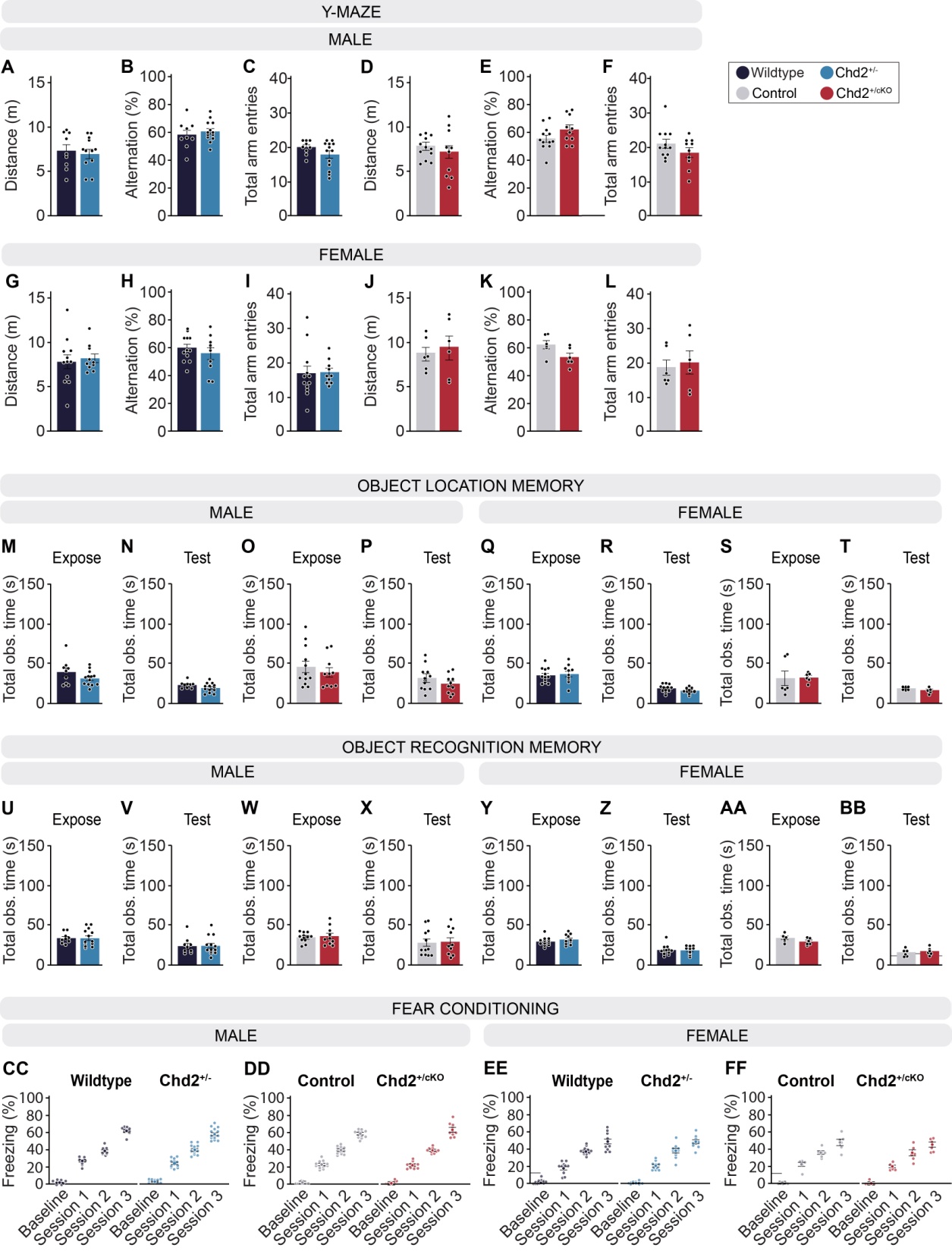
**

**Supplementary Figure 1.** Additional quantification of memory related behaviors in control and *Chd2* mutant mice. **A, D, G, J.** Quantification of total distance traveled in the y-maze. **B, E, H, K.** Spontaneous alternations during the test phase of the y-maze. **C, F, I, L.** Quantification of total number of arm entries. **M-T.** Total time spent exploring the objects during the exposure phase and test phase of object location memory test. **U-BB.** Total time spent exploring the objects during the exposure phase and test phase of object recognition memory test. **CC-FF.** Quantification of freezing behavior in context A before (baseline) and after each stimulation session during fear conditioning. See **Supplementary Table 3** for statistical analyses.


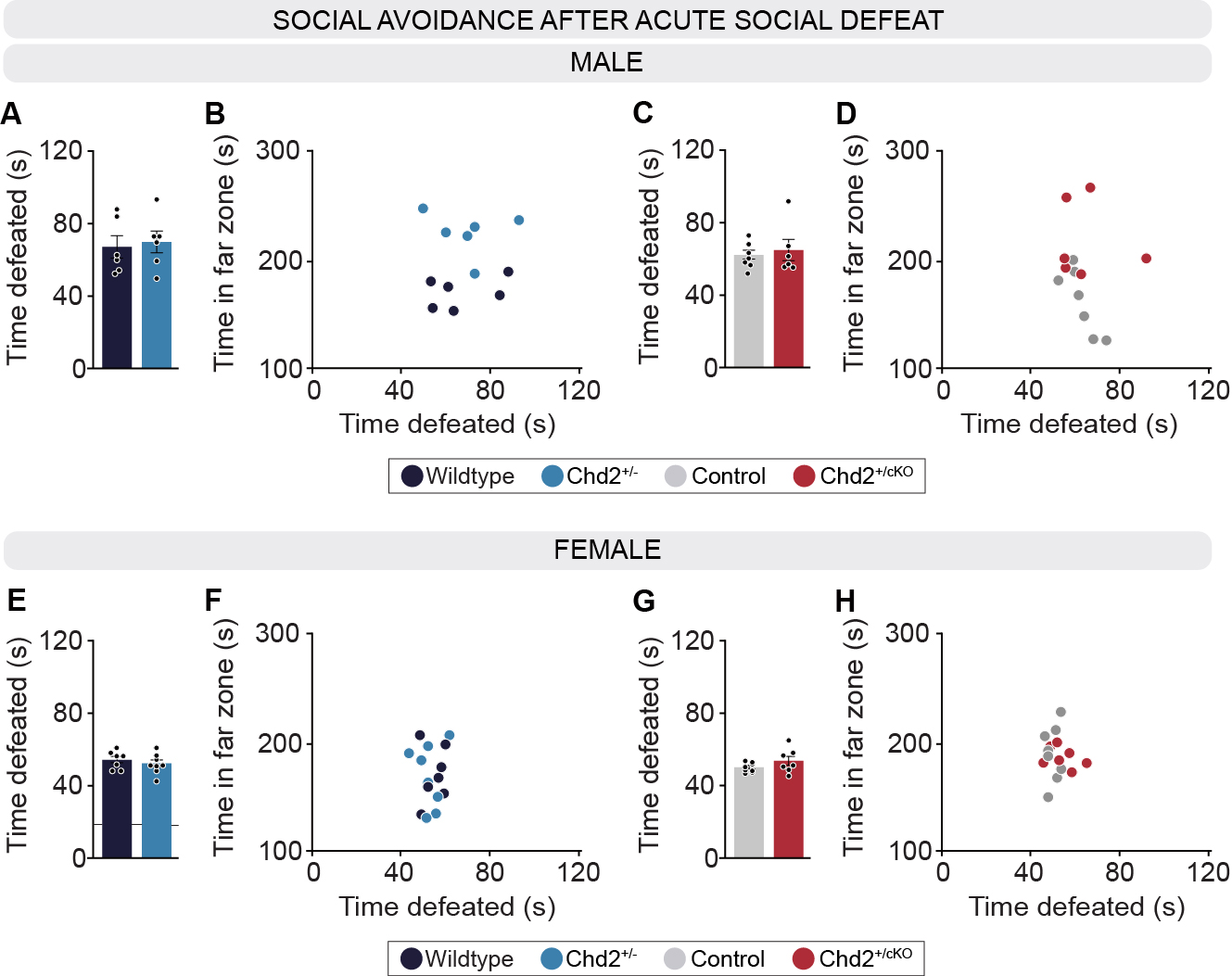


**Supplementary Figure 2.** Additional quantification of social avoidance in control and *Chd2* mutant mice. **A, C, E, G.** Total time spent defeated during exposure to acute social defeat. **B, D, F, H.** Scatter plots of time spent defeated versus time spent in far zone. See **Supplementary Table 3** for statistical analyses.


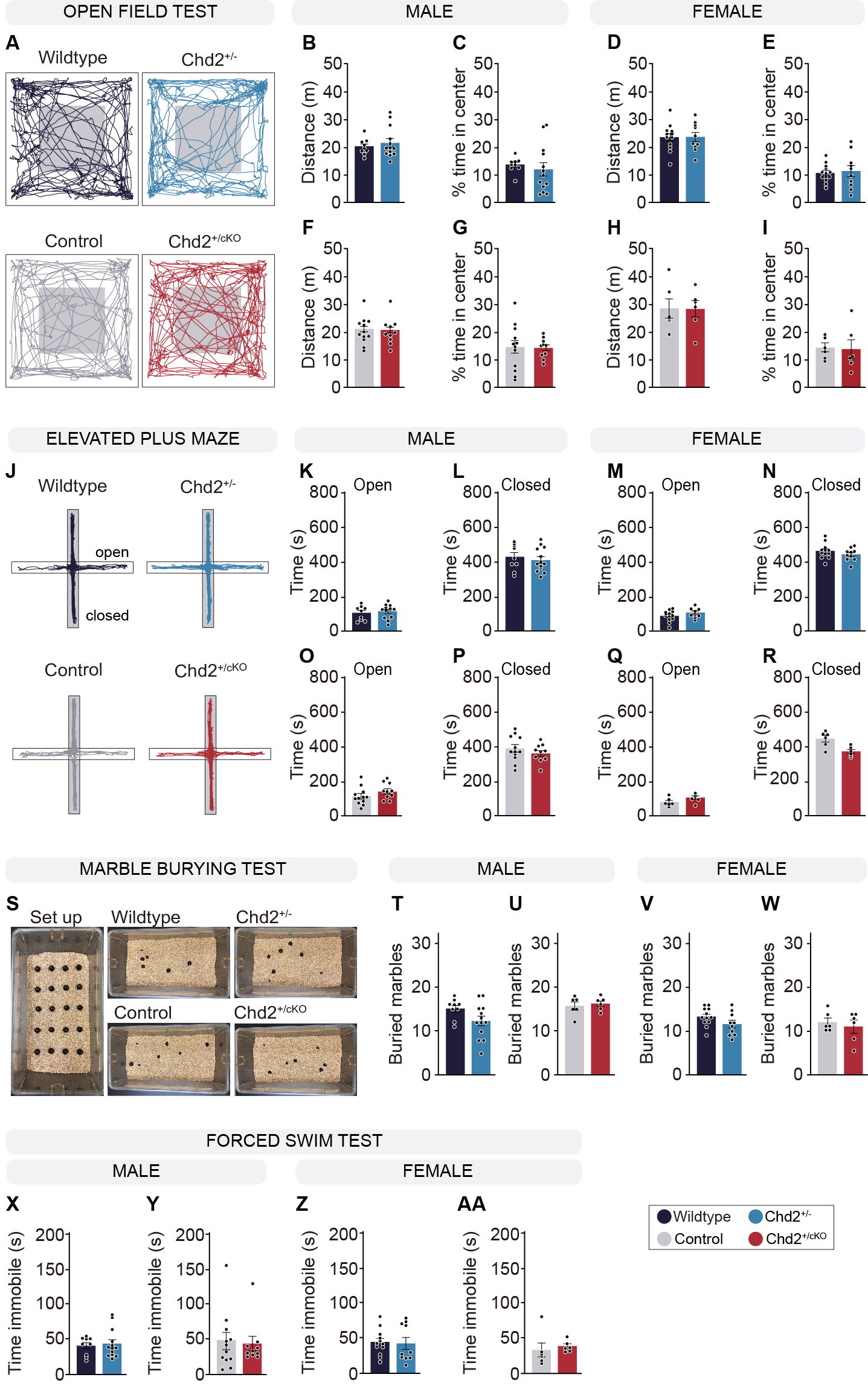


**Supplementary Figure 3.** General locomotor and anxiety-like behavior remains intact. **A.** Tracking plot of the paths taken by wildtype, *Chd2^+/-^*, control and *Chd2^+/cKO^* male mice in open field test. Center region is outlined by the inner square (grey). **B, D, F, H.** Total distance traveled in the open field. **C, E, G, I.** Percentage of time spent in the center of the arena. **J.** Representative tracking plots for a mouse in each group of the elevated plus maze. **K-R** Quantification of time spent in each portion of the arena. **S.** Example image of marbles before and after test. **T-W.** Quantification of buried marbles. **X-AA.** Time immobile during forced swim test. See **Supplementary Table 3** for statistical analyses.


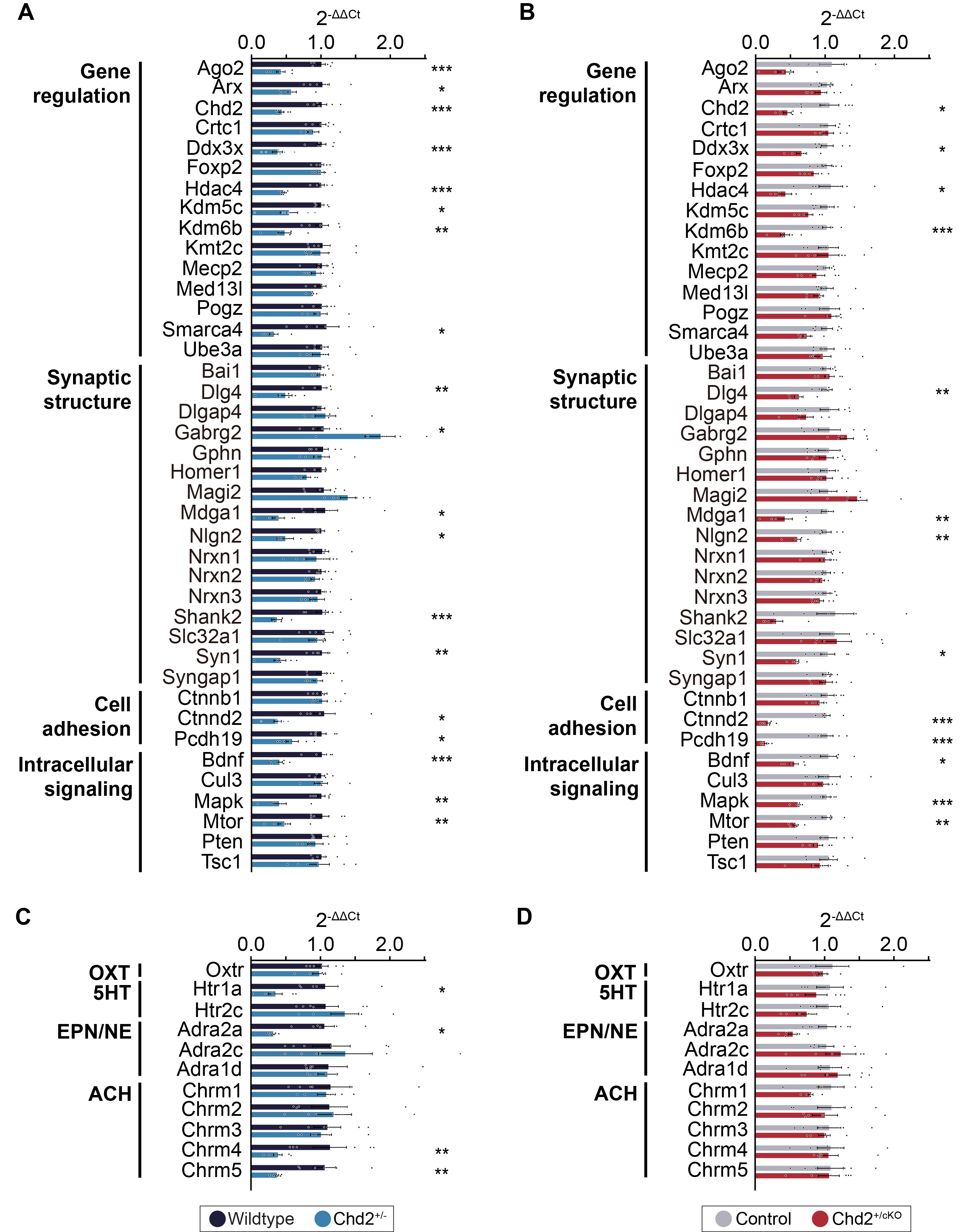


**Supplementary Figure 4.** Transcriptional changes in *Chd2^+/-^* and *Chd2^+/cKO^* mice. **A, C.** Quantification of gene expression in wildtype and *Chd2^+/-^* mice. **B, D.** Quantification of gene expression in control and *Chd2^+/cKO^* mice. *P < 0.05, **P < 0.01, ***P < 0.001, two-way repeated-measures ANOVA with Tukey’s *post hoc* test. See **Supplementary Table 2** for statistical analyses.


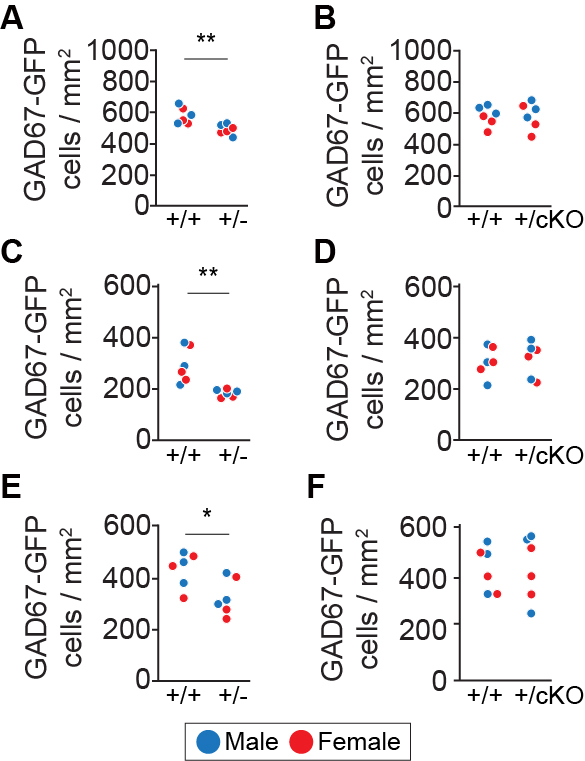


**Supplementary Figure 5. A-F.** Quantification of GAD67-GFP cell density in PFC (**A, B**), Hippocampus (**C, D**) and Amygdala (**E, F**) of male and female littermates.


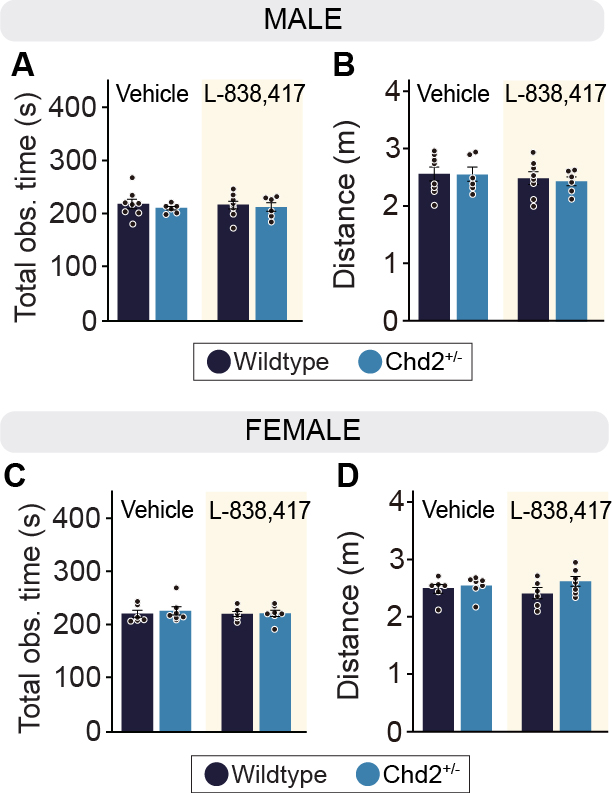


**Supplementary Figure 6. A, C.** Quantification of total time spent interacting with object or stranger of male (**A**) and female (**C**) wildtype and *Chd2^+/-^* mice. **B, D.** Quantification of total distance traveled during the test phase of male (**B**) and female (**D**) wildtype and *Chd2^+/-^* mice. See **Supplementary Table 3** for statistical analyses.

**Supplementary table 1. Primers for qPCR**

| **Gene name** | **Forward  (5'->3')** | **Reverse  (5'->3')** | **Reference** |
| --- | --- | --- | --- |
| Actb | GGCTGTATTCCCCTCCATCG | CCAGTTGGTAACAATGCCATGT | 15 |
| Adra1d | TGTCCCTAAATGTTCCCCAAG | CTATAAAGCACCCCACCTCTG | 56 |
| Adra2a | TTTCCCCTGTGCCTAACTGC | TGGCTTTATACACGGGGCTG | 57 |
| Adra2c | AGTTGCCAGAACCGCTCTTT | GAGCGCCTGAAGTCCTGATT | 57 |
| Ago2 | ACATTCCCGCAGGCACAA | GTCATCCCAAAGCACGTGGTAG | 58 |
| Arx | CCAAGGAAGAGCTGTTGCTGCAC | ACGGTTCTGGAACCACACCTGGAC | [59](https://anatomypubs.onlinelibrary.wiley.com/doi/10.1002/dvdy.20164) |
| Bai1 | TCTTCGGCTACTTCTCGGCGGC | CCTGTAGAAAGGCGAGGGGTGC | 60 |
| Bdnf | GGCGGCGCCCATGAAAGAAGT | GCCAGTGATGTCGTCGTCAGA | [61](https://www.pnas.org/doi/10.1073/pnas.2202015119#supplementary-materials) |
| Chd2 | CTTCTGAGAGCCAATCAGAGTC | CATCAGCTATCCGTTCCTTCTT | 15 |
| Chrm1 | TCTCTGAATGCTGGAAGTAAAGA | GAGACCCTAGATTCAGTCCCA | 62 |
| Chrm2 | AGTGTGGACAATTGGCTACTGG | ACCTTGTAGCGCCTATGTTCT | 63 |
| Chrm3 | AGGGCTGACTACTTAATCTTGGATA | TGCAAGGTCATTGTGACTCTC | 62 |
| Chrm4 | CCTGGTGACTGTGGTGGGTAACATCC | AACTGCCTGTTGACCTTGATGGACAG | 64 |
| Chrm5 | GAGATCATGACCAAGACATTGCCGACAA | TGGGAAGTCATTACTATTGCAGCTGTG | 64 |
| Crtc1 | CACCAGAGCACAATGACACCC | GCCTTCTTTGAGTCCCATGA | [65](https://pmc.ncbi.nlm.nih.gov/articles/PMC7900988/) |
| Ctnnb1 | CTGCTCATCCCACTAATGTC | CTTTATTAACTACCACCTGGTCCT | 66 |
| Ctnnd2 | GATGGCTCTGAGACGGAAACC | CTGGCTGGCTACGATCTGG | PrimerBank MGH-PGA |
| Cul3 | TCAAACAGTTGCAGCCAAAC | GAATCGAGCCTTCAGTTGCT | 67 |
| Ddx3x | CTCCGATTTCTCGGTACTCT | GACTTCCCTCTTGAATCACC | 68 |
| Dlg4 | ACCAGAAGAGTATAGCCGATTCG | GGTCTTGTCGTAGTCAAACAGG | 27 |
| Dlgap4 | TTTGCTTCTCTGCCCGATCC | TGATGAACATTGCTTCAAGAGC | 69 |
| FoxP2 | CCACGAAGACCTCAATGGTT | TCACGCTGAGGTTTCACAAG | [70](https://onlinelibrary.wiley.com/doi/10.1002/cne.10654) |
| Gabrg2 | ACTTCTGGTGACTATGTGGTGAT | GGCAGGAACAGCATCCTTATTG | 71 |
| Gapdh | AGGTCGGTGTGAACGGATTTG | TGTAGAACCATGTAGTTGAGGTCA | PrimerBank MGH-PGA |
| Gphn | ATACTGGCAGCCAGTAACTGGATAC | CAGCACTTGAGGAAGCATTGC | 72 |
| Hdac4 | CAATCCCACAGTCTCCGTGT | CAGCACCCCACTAAGGTTCA | 73 |
| Homer1 | AATGGTTAGGGGGCACTGTTT | CCCATCTGCCACAGTCACAA | [74](https://jneuroinflammation.biomedcentral.com/articles/10.1186/s12974-022-02428-8) |
| Htr1a | TGGATATGTTCAGTCTTGGCCAGG | ATTGCCGAGCACCGCGCAGAAAA | 75 |
| Htr2c | ATGGTGAACCTGGGCACTGCGG | CACGACTATTGAAAGTGCTGGCCA | 75 |
| Kdm5c | TTTGGCAGCGGTTTCCCTGTCAGT | AAGACCATTCCCACATACAGCC | 76 |
| Kdm6b | AGGAGTCACTGCAGGAGGAGAG | CCACCTCTTGGCATCAGACAG | 27 |
| Kmt2c | TGTTCACAGTGTGGTCAATGTT | GAGGGTCTAGGCAGTAGGTATG | [77](https://www.nature.com/articles/s41380-024-02479-8#Sec32) |
| Magi2 | CACCGCAAAGAAAACGAAGGG | TCCAATTTTATGGGGCACAG | 78 |
| Mapk | ACATGCACCATGAGAACGTCA | CTGGACCTTATCCTCGCTGAA | 79 |
| Mdga1 | AACGGTGCATCAGACAGTGA | TGGTGGCTGTGCAGTTGTAG | [80](https://www.thno.org/v15p1842.htm) |
| Mecp2 | CTCCATTATCCGTGACCGGG | AGCTTTTCCCTGGGGATTGA | 27 |
| Med13l | GACAGCACAAGCCCATCTGCAAAGT | ATGGCGGCAAACTTGAGCATAAAGT | 81 |
| Mtor | CACCAGAATTGGCAGATTTGC | CTTGGACGCCATTTCCATGAC | PrimerBank MGH-PGA |
| Nlgn 2 | CCATTTCCAGCTGGTCTGTCAACTAC | CCAAAGGCAATGTGGTAGCGGG | 82 |
| Nrxn 1 | ACTACATCAGTAACTCAGCACAG | ACAAGTGTCCGTTTCAAATCTTG | 83 |
| Nrxn 2 | GTCAGCAACAACTTCATGGG | AGCCACATCCTCACAACG | 83 |
| Nrxn 3 | GGGAGAACCTGCGAAAGAG | ATGAAGCGGAAGGACACATC | 83 |
| Oxtr | CCGCACAGTGAAGATGACCT | AGCATGGCAATGATGAAGGCAG | 84 |
| Pcdh19 | GCTTTGGTGTGCAGACCTAC | CGTCACCCCGCGTCTTTAT | PrimerBank MGH-PGA |
| Pogz | CTCTGCCTGCATGGCTTAGTA | CTGCAGACAAGTTCACGCAA | 27 |
| Pten | CCTGCAGAAAGACTTGAAGGTG | CTGTGCAACTCTGCAGTTAAA | 69 |
| Shank2 | TGCTGCCAGTGACTGCATTATTGA | CAGGGCTGGAAATGCTGGCGT | [85](https://jneurodevdisorders.biomedcentral.com/articles/10.1186/s11689-018-9233-1#Sec17) |
| Slc32a1 | GTCACGACAAACCCAAGATCAC | GGCGAAGATGATGAGGAACAAC | 86 |
| Smarca4 | AGATGGAGTAGCCCTTAGCA | GAGGTCCCCTCTCTAGACAGTT | 87 |
| Syn1 | TTCATGCCGCCTGTGGTAATGC | TGCTTCCCGACTCTTCTCTTGG | 88 |
| Syngap1 | CATCGAGCGAGAAGAGTACAAG | CCTCTCCTTCAGCGAATGTATC | [89](https://onlinelibrary.wiley.com/doi/10.1002/jgm.3717) |
| Tsc1 | TGCTCAGCCAGGTCTCTCAGA | CTCATTGACCTCCCCGAGCAC | 90 |
| UBE3A | CTCAAAGTCAGACGTGACCAT | CATCTACTCCTTGTTCTCCTTCA | [91](https://elifesciences.org/articles/81892#content) |

**Supplementary table 2. Statistics for qPCR analyses.**

| **Figure** | **Assay** | **No. of animals** | **Target gene** | **Reference gene** | **Group** | **Mean** | **SEM** | **Test** | **Statistics** | **Post-hoc test** | **Post-hoc statistics** |
| --- | --- | --- | --- | --- | --- | --- | --- | --- | --- | --- | --- |
| 1E | qPCR  (2^-ΔΔCT^) | WT = 6 Chd2^+/-^ = 7 | Chd2 | Actb | WT | 1.03 | 0.11 | two-tailed  t-test | t(11) = 4.120,  p = 0.000170, d = 2.29 |  |  |
|  |  |  |  |  | Chd2^+/-^ | 0.50 | 0.07 |  |  |  |  |
| 1F | qPCR  (2^-ΔΔCT^) | Control = 6 Chd2^+/cKO^ = 6 | Chd2 | Actb | Control | 0.93 | 0.06 | two-tailed  t-test | t(10) = 7.038,  p = 0.0000355, d = 4.06 |  |  |
|  |  |  |  |  | Chd2^+/cKO^ | 0.35 | 0.06 |  |  |  |  |
| S4A | qPCR  (2^-ΔΔCT^) | WT = 6 Chd2^+/-^ = 6 | Ago2 | Gapdh | WT | 1.01 | 0.06 | Two-Way repeat measured ANOVA | Genes F(7.721, 154.4) = 11.70 p = 1.18E-12  n^2^_p_ = 0.37  Genotype F(3,20) = 9.54 p = 0.00041 n^2^_p_ = 0.32  Interaction F(23.16, 154.4) = 5.024  p = 3.26E-10  n^2^_p_ = 0.43 | Tukey | WT vs Chd2^+/-^ p = 0.0002344 |
|  |  |  |  |  | Chd2^+/-^ | 0.42 | 0.06 |  |  |  |  |
|  |  |  | Arx | Gapdh | WT | 1.02 | 0.10 |  |  |  | WT vs Chd2^+/-^ p = 0.0213915 |
|  |  |  |  |  | Chd2^+/-^ | 0.57 | 0.08 |  |  |  |  |
|  |  |  | Chd2 | Gapdh | WT | 1.01 | 0.07 |  |  |  | WT vs Chd2^+/-^ p = 0.0005888 |
|  |  |  |  |  | Chd2^+/-^ | 0.43 | 0.04 |  |  |  |  |
|  |  |  | Crtc1 | Gapdh | WT | 1.01 | 0.06 |  |  |  | WT vs Chd2^+/-^ p = 0.6665948 |
|  |  |  |  |  | Chd2^+/-^ | 0.89 | 0.08 |  |  |  |  |
|  |  |  | Ddx3x | Gapdh | WT | 1.01 | 0.06 |  |  |  | WT vs Chd2^+/-^ p = 0.000308 |
|  |  |  |  |  | Chd2^+/-^ | 0.37 | 0.07 |  |  |  |  |
|  |  |  | Foxp2 | Gapdh | WT | 1.00 | 0.04 |  |  |  | WT vs Chd2^+/-^ p = 0.999942 |
|  |  |  |  |  | Chd2^+/-^ | 1.00 | 0.05 |  |  |  |  |
|  |  |  | Hdac4 | Gapdh | WT | 1.01 | 0.05 |  |  |  | WT vs Chd2^+/-^ p = 8.191E-05 |
|  |  |  |  |  | Chd2^+/-^ | 0.46 | 0.02 |  |  |  |  |
|  |  |  | Kdm5c | Gapdh | WT | 1.00 | 0.04 |  |  |  | WT vs Chd2^+/-^ p = 0.0466888 |
|  |  |  |  |  | Chd2^+/-^ | 0.54 | 0.13 |  |  |  |  |
|  |  |  | Kdm6b | Gapdh | WT | 1.02 | 0.09 |  |  |  | WT vs Chd2^+/-^ p = 0.0084297 |
|  |  |  |  |  | Chd2^+/-^ | 0.48 | 0.09 |  |  |  |  |
|  |  |  | Kmt2c | Gapdh | WT | 1.02 | 0.11 |  |  |  | WT vs Chd2^+/-^ p = 0.9969203 |
|  |  |  |  |  | Chd2^+/-^ | 0.99 | 0.11 |  |  |  |  |
|  |  |  | Mecp2 | Gapdh | WT | 1.02 | 0.07 |  |  |  | WT vs Chd2^+/-^ p = 0.8120571 |
|  |  |  |  |  | Chd2^+/-^ | 0.93 | 0.07 |  |  |  |  |
|  |  |  | Med13l | Gapdh | WT | 1.01 | 0.07 |  |  |  | WT vs Chd2^+/-^ p = 0.3019451 |
|  |  |  |  |  | Chd2^+/-^ | 0.87 | 0.02 |  |  |  |  |
|  |  |  | Pogz | Gapdh | WT | 1.01 | 0.07 |  |  |  | WT vs Chd2^+/-^ p = 0.9986714 |
|  |  |  |  |  | Chd2^+/-^ | 1.00 | 0.10 |  |  |  |  |
|  |  |  | Smarca4 | Gapdh | WT | 1.08 | 0.18 |  |  |  | WT vs Chd2^+/-^ p = 0.0289391 |
|  |  |  |  |  | Chd2^+/-^ | 0.32 | 0.06 |  |  |  |  |
|  |  |  | Ube3a | Gapdh | WT | 1.02 | 0.09 |  |  |  | WT vs Chd2^+/-^ p = 0.9987568 |
|  |  |  |  |  | Chd2^+/-^ | 0.99 | 0.12 |  |  |  |  |
|  |  |  | Bai1 | Gapdh | WT | 1.00 | 0.04 |  |  |  | WT vs Chd2^+/-^ p = 0.995982 |
|  |  |  |  |  | Chd2^+/-^ | 0.99 | 0.05 |  |  |  |  |
|  |  |  | Dlg4 | Gapdh | WT | 1.01 | 0.06 |  |  |  | WT vs Chd2^+/-^ p = 0.0079916 |
|  |  |  |  |  | Chd2^+/-^ | 0.49 | 0.10 |  |  |  |  |
|  |  |  | Dlgap4 | Gapdh | WT | 1.01 | 0.05 |  |  |  | WT vs Chd2^+/-^ p = 0.9781719 |
|  |  |  |  |  | Chd2^+/-^ | 1.07 | 0.15 |  |  |  |  |
|  |  |  | Gabrg2 | Gapdh | WT | 1.02 | 0.09 |  |  |  | WT vs Chd2^+/-^ p = 0.0442954 |
|  |  |  |  |  | Chd2^+/-^ | 1.82 | 0.21 |  |  |  |  |
|  |  |  | Gphn | Gapdh | WT | 1.01 | 0.08 |  |  |  | WT vs Chd2^+/-^ p = 0.9976116 |
|  |  |  |  |  | Chd2^+/-^ | 0.99 | 0.11 |  |  |  |  |
|  |  |  | Homer1 | Gapdh | WT | 1.01 | 0.07 |  |  |  | WT vs Chd2^+/-^ p = 0.1097401 |
|  |  |  |  |  | Chd2^+/-^ | 0.80 | 0.05 |  |  |  |  |
|  |  |  | Magi2 | Gapdh | WT | 1.02 | 0.10 |  |  |  | WT vs Chd2^+/-^ p = 0.1489709 |
|  |  |  |  |  | Chd2^+/-^ | 1.36 | 0.11 |  |  |  |  |
|  |  |  | Mdga1 | Gapdh | WT | 1.06 | 0.18 |  |  |  | WT vs Chd2^+/-^ p = 0.0471248 |
|  |  |  |  |  | Chd2^+/-^ | 0.39 | 0.09 |  |  |  |  |
|  |  |  | Nlgn2 | Gapdh | WT | 1.01 | 0.05 |  |  |  | WT vs Chd2^+/-^ p = 0.0215802 |
|  |  |  |  |  | Chd2^+/-^ | 0.49 | 0.12 |  |  |  |  |
|  |  |  | Nrxn1 | Gapdh | WT | 1.02 | 0.09 |  |  |  | WT vs Chd2^+/-^ p = 0.6645311 |
|  |  |  |  |  | Chd2^+/-^ | 0.92 | 0.17 |  |  |  |  |
|  |  |  | Nrxn2 | Gapdh | WT | 1.01 | 0.06 |  |  |  | WT vs Chd2^+/-^ p = 0.9371959 |
|  |  |  |  |  | Chd2^+/-^ | 0.85 | 0.06 |  |  |  |  |
|  |  |  | Nrxn3 | Gapdh | WT | 1.01 | 0.05 |  |  |  | WT vs Chd2^+/-^ p = 0.0729566 |
|  |  |  |  |  | Chd2^+/-^ | 0.96 | 0.11 |  |  |  |  |
|  |  |  | Shank2 | Gapdh | WT | 1.02 | 0.09 |  |  |  | WT vs Chd2^+/-^ p = 4.349E-05 |
|  |  |  |  |  | Chd2^+/-^ | 0.36 | 0.07 |  |  |  |  |
|  |  |  | Slc32a1 | Gapdh | WT | 1.04 | 0.12 |  |  |  | WT vs Chd2^+/-^ p = 0.9252685 |
|  |  |  |  |  | Chd2^+/-^ | 0.93 | 0.12 |  |  |  |  |
|  |  |  | Syn1 | Gapdh | WT | 1.02 | 0.09 |  |  |  | WT vs Chd2^+/-^ p = 0.0025367 |
|  |  |  |  |  | Chd2^+/-^ | 0.41 | 0.09 |  |  |  |  |
|  |  |  | Syngap1 | Gapdh | WT | 1.01 | 0.07 |  |  |  | WT vs Chd2^+/-^ p = 0.9165181 |
|  |  |  |  |  | Chd2^+/-^ | 0.95 | 0.08 |  |  |  |  |
|  |  |  | Ctnnb1 | Gapdh | WT | 1.01 | 0.08 |  |  |  | WT vs Chd2^+/-^ p = 0.999961 |
|  |  |  |  |  | Chd2^+/-^ | 1.02 | 0.07 |  |  |  |  |
|  |  |  | Ctnnd2 | Gapdh | WT | 1.05 | 0.15 |  |  |  | WT vs Chd2^+/-^ p = 0.0219562 |
|  |  |  |  |  | Chd2^+/-^ | 0.38 | 0.05 |  |  |  |  |
|  |  |  | Pcdh19 | Gapdh | WT | 1.01 | 0.07 |  |  |  | WT vs Chd2^+/-^ p = 0.0166212 |
|  |  |  |  |  | Chd2^+/-^ | 0.58 | 0.09 |  |  |  |  |
|  |  |  | Bdnf | Gapdh | WT | 1.01 | 0.07 |  |  |  | WT vs Chd2^+/-^ p = 0.0001995 |
|  |  |  |  |  | Chd2^+/-^ | 0.40 | 0.04 |  |  |  |  |
|  |  |  | Cul3 | Gapdh | WT | 1.01 | 0.05 |  |  |  | WT vs Chd2^+/-^ p = 0.9999625 |
|  |  |  |  |  | Chd2^+/-^ | 1.00 | 0.10 |  |  |  |  |
|  |  |  | Mapk | Gapdh | WT | 1.01 | 0.05 |  |  |  | WT vs Chd2^+/-^ p = 0.0043401 |
|  |  |  |  |  | Chd2^+/-^ | 0.40 | 0.10 |  |  |  |  |
|  |  |  | Mtor | Gapdh | WT | 1.02 | 0.10 |  |  |  | WT vs Chd2^+/-^ p = 0.0086778 |
|  |  |  |  |  | Chd2^+/-^ | 0.47 | 0.09 |  |  |  |  |
|  |  |  | Pten | Gapdh | WT | 1.02 | 0.09 |  |  |  | WT vs Chd2^+/-^ p = 0.9089365 |
|  |  |  |  |  | Chd2^+/-^ | 0.92 | 0.11 |  |  |  |  |
|  |  |  | Tsc1 | Gapdh | WT | 1.01 | 0.06 |  |  |  | WT vs Chd2^+/-^ p = 0.9939324 |
|  |  |  |  |  | Chd2^+/-^ | 0.97 | 0.16 |  |  |  |  |
| S4B | qPCR  (2^-ΔΔCT^) | Control = 6 Chd2^+/cKO^ = 6 | Ago2 | Gapdh | Control | 1.08 | 0.18 |  |  |  | Ctrl vs Chd2^+/cKO^ p = 0.0518589 |
|  |  |  |  |  | Chd2^+/cKO^ | 0.42 | 0.11 |  |  |  |  |
|  |  |  | Arx | Gapdh | Control | 1.02 | 0.08 |  |  |  | Ctrl vs Chd2^+/cKO^ p = 0.8781408 |
|  |  |  |  |  | Chd2^+/cKO^ | 0.93 | 0.08 |  |  |  |  |
|  |  |  | Chd2 | Gapdh | Control | 1.05 | 0.14 |  |  |  | Ctrl vs Chd2^+/cKO^ p = 0.0206304 |
|  |  |  |  |  | Chd2^+/cKO^ | 0.44 | 0.06 |  |  |  |  |
|  |  |  | Crtc1 | Gapdh | Control | 1.03 | 0.11 |  |  |  | Ctrl vs Chd2^+/cKO^ p = 0.9999992 |
|  |  |  |  |  | Chd2^+/cKO^ | 1.03 | 0.07 |  |  |  |  |
|  |  |  | Ddx3x | Gapdh | Control | 1.02 | 0.09 |  |  |  | Ctrl vs Chd2^+/cKO^ p = 0.0385143 |
|  |  |  |  |  | Chd2^+/cKO^ | 0.65 | 0.07 |  |  |  |  |
|  |  |  | Foxp2 | Gapdh | Control | 1.01 | 0.07 |  |  |  | Ctrl vs Chd2^+/cKO^ p = 0.2487745 |
|  |  |  |  |  | Chd2^+/cKO^ | 0.82 | 0.06 |  |  |  |  |
|  |  |  | Hdac4 | Gapdh | Control | 1.07 | 0.17 |  |  |  | Ctrl vs Chd2^+/cKO^ p = 0.0409355 |
|  |  |  |  |  | Chd2^+/cKO^ | 0.42 | 0.09 |  |  |  |  |
|  |  |  | Kdm5c | Gapdh | Control | 1.02 | 0.10 |  |  |  | Ctrl vs Chd2^+/cKO^ p = 0.1623623 |
|  |  |  |  |  | Chd2^+/cKO^ | 0.74 | 0.07 |  |  |  |  |
|  |  |  | Kdm6b | Gapdh | Control | 1.01 | 0.06 |  |  |  | Ctrl vs Chd2^+/cKO^ p = 0.000324 |
|  |  |  |  |  | Chd2^+/cKO^ | 0.41 | 0.07 |  |  |  |  |
|  |  |  | Kmt2c | Gapdh | Control | 1.04 | 0.14 |  |  |  | Ctrl vs Chd2^+/cKO^ p = 0.9999753 |
|  |  |  |  |  | Chd2^+/cKO^ | 1.03 | 0.15 |  |  |  |  |
|  |  |  | Mecp2 | Gapdh | Control | 1.01 | 0.05 |  |  |  | Ctrl vs Chd2^+/cKO^ p = 0.1069383 |
|  |  |  |  |  | Chd2^+/cKO^ | 0.70 | 0.10 |  |  |  |  |
|  |  |  | Med13l | Gapdh | Control | 1.02 | 0.09 |  |  |  | Ctrl vs Chd2^+/cKO^ p = 0.7064851 |
|  |  |  |  |  | Chd2^+/cKO^ | 0.90 | 0.07 |  |  |  |  |
|  |  |  | Pogz | Gapdh | Control | 1.05 | 0.14 |  |  |  | Ctrl vs Chd2^+/cKO^ p = 0.9986578 |
|  |  |  |  |  | Chd2^+/cKO^ | 1.08 | 0.08 |  |  |  |  |
|  |  |  | Smarca4 | Gapdh | Control | 1.02 | 0.08 |  |  |  | Ctrl vs Chd2^+/cKO^ p = 0.0582515 |
|  |  |  |  |  | Chd2^+/cKO^ | 0.72 | 0.06 |  |  |  |  |
|  |  |  | Ube3a | Gapdh | Control | 1.02 | 0.09 |  |  |  | Ctrl vs Chd2^+/cKO^ p = 0.969233 |
|  |  |  |  |  | Chd2^+/cKO^ | 0.96 | 0.12 |  |  |  |  |
|  |  |  | Bai1 | Gapdh | Control | 1.01 | 0.06 |  |  |  | Ctrl vs Chd2^+/cKO^ p = 0.9634339 |
|  |  |  |  |  | Chd2^+/cKO^ | 1.05 | 0.07 |  |  |  |  |
|  |  |  | Dlg4 | Gapdh | Control | 1.01 | 0.08 |  |  |  | Ctrl vs Chd2^+/cKO^ p = 0.0091288 |
|  |  |  |  |  | Chd2^+/cKO^ | 0.61 | 0.06 |  |  |  |  |
|  |  |  | Dlgap4 | Gapdh | Control | 1.05 | 0.14 |  |  |  | Ctrl vs Chd2^+/cKO^ p = 0.260633 |
|  |  |  |  |  | Chd2^+/cKO^ | 0.71 | 0.10 |  |  |  |  |
|  |  |  | Gabrg2 | Gapdh | Control | 1.06 | 0.16 |  |  |  | Ctrl vs Chd2^+/cKO^ p = 0.5520904 |
|  |  |  |  |  | Chd2^+/cKO^ | 1.31 | 0.10 |  |  |  |  |
|  |  |  | Gphn | Gapdh | Control | 1.05 | 0.16 |  |  |  | Ctrl vs Chd2^+/cKO^ p = 0.9966057 |
|  |  |  |  |  | Chd2^+/cKO^ | 1.01 | 0.10 |  |  |  |  |
|  |  |  | Homer1 | Gapdh | Control | 1.03 | 0.11 |  |  |  | Ctrl vs Chd2^+/cKO^ p = 0.9984839 |
|  |  |  |  |  | Chd2^+/cKO^ | 1.01 | 0.09 |  |  |  |  |
|  |  |  | Magi2 | Gapdh | Control | 1.04 | 0.13 |  |  |  | Ctrl vs Chd2^+/cKO^ p = 0.1983504 |
|  |  |  |  |  | Chd2^+/cKO^ | 1.45 | 0.14 |  |  |  |  |
|  |  |  | Mdga1 | Gapdh | Control | 1.02 | 0.09 |  |  |  | Ctrl vs Chd2^+/cKO^ p = 0.0072058 |
|  |  |  |  |  | Chd2^+/cKO^ | 0.41 | 0.11 |  |  |  |  |
|  |  |  | Nlgn2 | Gapdh | Control | 1.01 | 0.06 |  |  |  | Ctrl vs Chd2^+/cKO^ p = 0.0023349 |
|  |  |  |  |  | Chd2^+/cKO^ | 0.59 | 0.05 |  |  |  |  |
|  |  |  | Nrxn1 | Gapdh | Control | 1.01 | 0.07 |  |  |  | Ctrl vs Chd2^+/cKO^ p = 0.991929 |
|  |  |  |  |  | Chd2^+/cKO^ | 0.98 | 0.08 |  |  |  |  |
|  |  |  | Nrxn2 | Gapdh | Control | 1.01 | 0.06 |  |  |  | Ctrl vs Chd2^+/cKO^ p = 0.8815438 |
|  |  |  |  |  | Chd2^+/cKO^ | 0.76 | 0.04 |  |  |  |  |
|  |  |  | Nrxn3 | Gapdh | Control | 1.01 | 0.05 |  |  |  | Ctrl vs Chd2^+/cKO^ p = 0.6006552 |
|  |  |  |  |  | Chd2^+/cKO^ | 0.91 | 0.05 |  |  |  |  |
|  |  |  | Shank2 | Gapdh | Control | 1.30 | 0.41 |  |  |  | Ctrl vs Chd2^+/cKO^ p = 0.1786844 |
|  |  |  |  |  | Chd2^+/cKO^ | 0.28 | 0.10 |  |  |  |  |
|  |  |  | Slc32a1 | Gapdh | Control | 1.13 | 0.21 |  |  |  | Ctrl vs Chd2^+/cKO^ p = 0.9252685 |
|  |  |  |  |  | Chd2^+/cKO^ | 1.16 | 0.21 |  |  |  |  |
|  |  |  | Syn1 | Gapdh | Control | 1.02 | 0.10 |  |  |  | Ctrl vs Chd2^+/cKO^ p = 0.0191284 |
|  |  |  |  |  | Chd2^+/cKO^ | 0.57 | 0.04 |  |  |  |  |
|  |  |  | Syngap1 | Gapdh | Control | 1.01 | 0.06 |  |  |  | Ctrl vs Chd2^+/cKO^ p = 0.9999123 |
|  |  |  |  |  | Chd2^+/cKO^ | 1.00 | 0.09 |  |  |  |  |
|  |  |  | Ctnnb1 | Gapdh | Control | 1.02 | 0.09 |  |  |  | Ctrl vs Chd2^+/cKO^ p = 0.7367122 |
|  |  |  |  |  | Chd2^+/cKO^ | 0.91 | 0.06 |  |  |  |  |
|  |  |  | Ctnnd2 | Gapdh | Control | 1.01 | 0.05 |  |  |  | Ctrl vs Chd2^+/cKO^ p = 2.36E-06 |
|  |  |  |  |  | Chd2^+/cKO^ | 0.16 | 0.04 |  |  |  |  |
|  |  |  | Pcdh19 | Gapdh | Control | 1.02 | 0.08 |  |  |  | Ctrl vs Chd2^+/cKO^ p = 0.000246 |
|  |  |  |  |  | Chd2^+/cKO^ | 0.13 | 0.02 |  |  |  |  |
|  |  |  | Bdnf | Gapdh | Control | 1.03 | 0.12 |  |  |  | Ctrl vs Chd2^+/cKO^ p = 0.0244224 |
|  |  |  |  |  | Chd2^+/cKO^ | 0.54 | 0.06 |  |  |  |  |
|  |  |  | Cul3 | Gapdh | Control | 1.05 | 0.15 |  |  |  | Ctrl vs Chd2^+/cKO^ p = 0.9489408 |
|  |  |  |  |  | Chd2^+/cKO^ | 0.96 | 0.07 |  |  |  |  |
|  |  |  | Mapk | Gapdh | Control | 1.01 | 0.05 |  |  |  | Ctrl vs Chd2^+/cKO^ p = 0.000936 |
|  |  |  |  |  | Chd2^+/cKO^ | 0.60 | 0.03 |  |  |  |  |
|  |  |  | Mtor | Gapdh | Control | 1.01 | 0.07 |  |  |  | Ctrl vs Chd2^+/cKO^ p = 0.0025633 |
|  |  |  |  |  | Chd2^+/cKO^ | 0.56 | 0.03 |  |  |  |  |
|  |  |  | Pten | Gapdh | Control | 1.04 | 0.12 |  |  |  | Ctrl vs Chd2^+/cKO^ p = 0.6773552 |
|  |  |  |  |  | Chd2^+/cKO^ | 0.89 | 0.07 |  |  |  |  |
|  |  |  | Tsc1 | Gapdh | Control | 1.04 | 0.13 |  |  |  | Ctrl vs Chd2^+/cKO^ p = 0.8876327 |
|  |  |  |  |  | Chd2^+/cKO^ | 0.91 | 0.12 |  |  |  |  |
| S4C | qPCR  (2^-ΔΔCT^) | WT = 6 Chd2^+/-^ = 6 | Oxtr | Gapdh | WT | 1.02 | 0.09 | Two-Way repeat measured ANOVA | Genes F(6.513, 130.3) = 4.81 p = 0.0001  n^2^_p_ = 0.19  Genotype F(3,20) = 2.101 p = 0.0322  n^2^_p_ = 0.16  Interaction F(19.54, 130.3) = 2.591  p = 0.0007  n^2^_p_ = 0.28 | Tukey | WT vs Chd2^+/-^ p = 0.9995 |
|  |  |  |  |  | Chd2^+/-^ | 0.98 | 0.09 |  |  |  |  |
|  |  |  | Htr1a | Gapdh | WT | 1.06 | 0.18 |  |  |  | WT vs Chd2^+/-^ p = 0.0175 |
|  |  |  |  |  | Chd2^+/-^ | 0.35 | 0.10 |  |  |  |  |
|  |  |  | Htr2c | Gapdh | WT | 1.07 | 0.18 |  |  |  | WT vs Chd2^+/-^ p = 0.9911 |
|  |  |  |  |  | Chd2^+/-^ | 1.34 | 0.20 |  |  |  |  |
|  |  |  | Adra2a | Gapdh | WT | 1.05 | 0.14 |  |  |  | WT vs Chd2^+/-^ p = 0.0121 |
|  |  |  |  |  | Chd2^+/-^ | 0.32 | 0.02 |  |  |  |  |
|  |  |  | Adra2c | Gapdh | WT | 1.15 | 0.27 |  |  |  | WT vs Chd2^+/-^ p = 0.7624 |
|  |  |  |  |  | Chd2^+/-^ | 1.35 | 0.38 |  |  |  |  |
|  |  |  | Adra1d | Gapdh | WT | 1.11 | 0.27 |  |  |  | WT vs Chd2^+/-^ p = 0.988 |
|  |  |  |  |  | Chd2^+/-^ | 1.09 | 0.14 |  |  |  |  |
|  |  |  | Chrm1 | Gapdh | WT | 1.14 | 0.28 |  |  |  | WT vs Chd2^+/-^ p = 0.8493 |
|  |  |  |  |  | Chd2^+/-^ | 1.08 | 0.13 |  |  |  |  |
|  |  |  | Chrm2 | Gapdh | WT | 1.12 | 0.25 |  |  |  | WT vs Chd2^+/-^ p = 0.9933 |
|  |  |  |  |  | Chd2^+/-^ | 1.18 | 0.26 |  |  |  |  |
|  |  |  | Chrm3 | Gapdh | WT | 1.10 | 0.19 |  |  |  | WT vs Chd2^+/-^ p = 0.9995 |
|  |  |  |  |  | Chd2^+/-^ | 1.00 | 0.15 |  |  |  |  |
|  |  |  | Chrm4 | Gapdh | WT | 1.13 | 0.24 |  |  |  | WT vs Chd2^+/-^ p = 0.0087 |
|  |  |  |  |  | Chd2^+/-^ | 0.39 | 0.06 |  |  |  |  |
|  |  |  | Chrm5 | Gapdh | WT | 1.05 | 0.16 |  |  |  | WT vs Chd2^+/-^ p = 0.0037 |
|  |  |  |  |  | Chd2^+/-^ | 0.38 | 0.02 |  |  |  |  |
| S4D | qPCR  (2^-ΔΔCT^) | Control = 6 Chd2^+/cKO^ = 6 | Oxtr | Gapdh | Control | 1.11 | 0.24 |  |  |  | Ctrl vs Chd2^+/cKO^ p = 0.9323 |
|  |  |  |  |  | Chd2^+/cKO^ | 0.98 | 0.06 |  |  |  |  |
|  |  |  | Htr1a | Gapdh | Control | 1.08 | 0.19 |  |  |  | Ctrl vs Chd2^+/cKO^ p = 0.753 |
|  |  |  |  |  | Chd2^+/cKO^ | 0.88 | 0.16 |  |  |  |  |
|  |  |  | Htr2c | Gapdh | Control | 1.06 | 0.17 |  |  |  | Ctrl vs Chd2^+/cKO^ p = 0.9799 |
|  |  |  |  |  | Chd2^+/cKO^ | 0.74 | 0.14 |  |  |  |  |
|  |  |  | Adra2a | Gapdh | Control | 1.04 | 0.13 |  |  |  | Ctrl vs Chd2^+/cKO^ p = 0.0608 |
|  |  |  |  |  | Chd2^+/cKO^ | 0.54 | 0.06 |  |  |  |  |
|  |  |  | Adra2c | Gapdh | Control | 1.03 | 0.11 |  |  |  | Ctrl vs Chd2^+/cKO^ p = 0.9986 |
|  |  |  |  |  | Chd2^+/cKO^ | 1.23 | 0.22 |  |  |  |  |
|  |  |  | Adra1d | Gapdh | Control | 1.08 | 0.17 |  |  |  | Ctrl vs Chd2^+/cKO^ p = 0.8493 |
|  |  |  |  |  | Chd2^+/cKO^ | 1.19 | 0.18 |  |  |  |  |
|  |  |  | Chrm1 | Gapdh | Control | 1.09 | 0.18 |  |  |  | Ctrl vs Chd2^+/cKO^ p = 0.8351 |
|  |  |  |  |  | Chd2^+/cKO^ | 0.80 | 0.04 |  |  |  |  |
|  |  |  | Chrm2 | Gapdh | Control | 1.10 | 0.20 |  |  |  | Ctrl vs Chd2^+/cKO^ p = 0.9153 |
|  |  |  |  |  | Chd2^+/cKO^ | 1.01 | 0.18 |  |  |  |  |
|  |  |  | Chrm3 | Gapdh | Control | 1.07 | 0.17 |  |  |  | Ctrl vs Chd2^+/cKO^ p = 0.9852 |
|  |  |  |  |  | Chd2^+/cKO^ | 1.00 | 0.08 |  |  |  |  |
|  |  |  | Chrm4 | Gapdh | Control | 1.08 | 0.19 |  |  |  | Ctrl vs Chd2^+/cKO^ p = 0.7743 |
|  |  |  |  |  | Chd2^+/cKO^ | 1.06 | 0.14 |  |  |  |  |
|  |  |  | Chrm5 | Gapdh | Control | 1.09 | 0.19 |  |  |  | Ctrl vs Chd2^+/cKO^ p = 0.7374 |
|  |  |  |  |  | Chd2^+/cKO^ | 1.06 | 0.15 |  |  |  |  |

**Supplementary table 3. Statistics for behavior tests.**

| **Fig** | **Assay** | **No. of animals** | **Measurement** | | **Group** | | **Mean** | **SEM** | **Test** | **Statistics** | **Post-hoc** | **Post-hoc statistics** |
| --- | --- | --- | --- | --- | --- | --- | --- | --- | --- | --- | --- | --- |
| 1G | Weight male | WT = 20 Chd2^+/-^ = 19 | Weight  (g) | P55 | WT | | 21.56 | 0.14 | Two-way  RM ANOVA | Genotype, F (1, 37) = 135.9, p = 6E-14, η²_p_ = 0.78 Time, F (1, 37) = 1304, p = 1.02E-15, η²_p_ = 0.97 Interaction, F (1, 37) = 73.41, p = 2.59E-10, η²_p_ = 0.66 | Tukey | P55, WT vs Chd2^+/-^, p = 9.83E-09 P83, WT vs Chd2^+/-^, p = 1.05E-15 WT, P55 vs P83, p = 1.02E-15 Chd2^+/-^, P55 vs P83, p = 1.47E-14 |
|  |  |  |  |  | Chd2^+/-^ | | 19.45 | 0.29 |  |  |  |  |
|  |  |  |  | P83 | WT | | 28.24 | 0.21 |  |  |  |  |
|  |  |  |  |  | Chd2^+/-^ | | 23.57 | 0.27 |  |  |  |  |
| 1G |  | Control = 23 Chd2^+/cKO^ = 21 | Weight  (g) | P55 | Control | | 21.54 | 0.12 | Two-way  RM ANOVA | Genotype, F (1, 42) = 0.3730, p = 0.54, η²_p_ = 0.0088 Time, F (1, 42) = 2224, p = 1.04E-15, η²_p_ = 0.98 Interaction, F (1, 42) = 1.169, p = 0.29, η²_p_ = 0.027 | Tukey | P55, WT vs Chd2^+/cKO^, p = 0.88 P83, WT vs Chd2^+/cKO^, p = 0.26 WT, P55 vs P83, p = 1.04E-13 Chd2^+/cKO^, P55 vs P83, p = 1.55E-13 |
|  |  |  |  |  | Chd2^+/cKO^ | | 21.57 | 0.14 |  |  |  |  |
|  |  |  |  | P83 | Control | | 27.84 | 0.14 |  |  |  |  |
|  |  |  |  |  | Chd2^+/cKO^ | | 27.59 | 0.21 |  |  |  |  |
| 1H | Weight  female | WT = 11 Chd2^+/-^ = 13 | Weight  (g) | P55 | WT | | 18.84 | 0.26 | Two-way  RM ANOVA | Genotype, F (1, 22) = 42.27, p = 1.54E-06, η²_p_ = 0.66 Time, F (1, 22) = 979.6, p = 1.08E-15, η²_p_ = 0.98 Interaction, F (1, 22) = 0.9648, p = 0.34,η²_p_ = 0.042 | Tukey | P55, WT vs Chd2^+/-^, p = 3.27E-07 P83, WT vs Chd2^+/-^, p = 5.62E-08 WT, P55 vs P83, p = 1.07E-15 Chd2^+/-^, P55 vs P83, p = 1.01E-15 |
|  |  |  |  |  | Chd2^+/-^ | | 16.56 | 0.22 |  |  |  |  |
|  |  |  |  | P83 | WT | | 22.09 | 0.39 |  |  |  |  |
|  |  |  |  |  | Chd2^+/-^ | | 19.61 | 0.20 |  |  |  |  |
| 1H |  | Control= 9 Chd2^+/cKO^ = 8 | Weight  (g) | P55 | Control | | 18.71 | 0.24 | Two-way  RM ANOVA | Genotype, F (1, 15) = 2.916, p = 0.11, η²_p_ = 0.16 Time, F (1, 15) = 2353, p = 1.11E-15, η²_p_ = 0.99 Interaction, F (1, 15) = 0.6189, p = 0.44, η²_p_ = 0.0396 | Tukey | P55, WT vs Chd2^+/cKO^, p = 0.077 P83, WT vs Chd2^+/cKO^, p = 0.14 WT, P55 vs P83, p = 1.05E-15 Chd2^+/cKO^, P55 vs P83, p = 2E-15 |
|  |  |  |  |  | Chd2^+/cKO^ | | 19.31 | 0.17 |  |  |  |  |
|  |  |  |  | P83 | Control | | 21.91 | 0.29 |  |  |  |  |
|  |  |  |  |  | Chd2^+/cKO^ | | 22.41 | 0.16 |  |  |  |  |
| 2B | Y maze male | WT = 9 Chd2^+/-^ = 13 | Time in arm (s) | | WT | Familiar | 71.80 | 6.46 | Two-way  ANOVA | Arm, F (2.54) = 6.42, p = 0.0032, η²_p_ = 0.19 Genotype, F (1, 54) = 1.48E-07, p = 0.9997,  η²_p_ = 2.75E-15 Interaction, F (2, 54) = 24.51, p = 2.67E-08, η²_p_ = 0.48 | Tukey | Familiar vs entry, p = 0.999992 Familiar vs Novel, p = 0.000252 Entry vs Novel, p = 0.000403 |
|  |  |  |  |  |  | Entry | 74.22 | 9.54 |  |  |  |  |
|  |  |  |  |  |  | Novel | 153.99 | 9.61 |  |  |  |  |
|  |  |  |  |  | Chd2^+/-^ | Familiar | 75.22 | 10.91 |  |  | Tukey | Familiar vs entry, p = 0.000337 Familiar vs Novel, p = 0.976 Entry vs Novel, p = 0.0000258 |
|  |  |  |  |  |  | Entry | 153.22 | 14.62 |  |  |  |  |
|  |  |  |  |  |  | Novel | 71.55 | 9.15 |  |  |  |  |
| 2C |  | WT = 9 Chd2^+/-^ = 13 | Discrimination index | | WT | | 36.11 | 5.31 | Two-tailed  t-test | t(20) = 3.25,  p = 0.004, d = 1.38 |  |  |
|  |  |  |  |  | Chd2^+/-^ | | 0.15 | 8.39 |  |  |  |  |
| 2D |  | Control = 12 Chd2^+/cKO^ = 10 | Time in arm (s) | | Control | Familiar | 73.15 | 3.92 | Two-way  ANOVA | Arm, F (2, 60) = 12.09, p = 3.86E-05, η²_p_ = 0.28 Genotype, F (1, 60) = 0.025, p = 0.99, η²_p_ = 0.02 Interaction, F (2, 60) = 18.54, p = 5.38E-07, η²_p_ = 0.38 | Tukey | Familiar vs entry, p = 0.67 Familiar vs Novel, p = 1.41E-08 Entry vs Novel, p = 4.64E-06 |
|  |  |  |  |  |  | Entry | 86.93 | 5.63 |  |  |  |  |
|  |  |  |  |  |  | Novel | 139.92 | 6.47 |  |  |  |  |
|  |  |  |  |  | Chd2^+/cKO^ | Familiar | 91.96 | 5.65 |  |  | Tukey | Familiar vs entry, p = 0.19 Familiar vs Novel, p = 0.99 Entry vs Novel, p = 0.2 |
|  |  |  |  |  |  | Entry | 115.81 | 9.50 |  |  |  |  |
|  |  |  |  |  |  | Novel | 92.23 | 9.32 |  |  |  |  |
| 2E |  | Control = 12 Chd2^+/cKO^ = 10 | Discrimination index | | Control | | 30.96 | 3.72 | Two-tailed  t-test | t(20) = 4.24,  p = 0.00041, d = 1.81 |  |  |
|  |  |  |  |  | Chd2^+/cKO^ | | -1.23 | 7.05 |  |  |  |  |
| 2G | Y-maze  female | WT = 12 Chd2^+/-^ = 10 | Time in arm (s) | | WT | Familiar | 75.68 | 7.79 | Two-way  ANOVA | Arm, F (2, 60) = 6.135, p = 0.0038, η²_p_ = 0.17 Genotype, F (1, 60) = 4.302E-07, p = 0.99,  η²_p_ = 7.17E-09 Interaction, F (2, 60) = 15.14, p = 4.75E-06, η²_p_ = 0.33 | Tukey | Familiar vs entry, p = 0.99 Familiar vs Novel, p = 0.000024 Entry vs Novel, p = 0.0000028 |
|  |  |  |  |  |  | Entry | 66.91 | 9.13 |  |  |  |  |
|  |  |  |  |  |  | Novel | 157.42 | 12.10 |  |  |  |  |
|  |  |  |  |  | Chd2^+/-^ | Familiar | 93.30 | 14.61 |  |  | Tukey | Familiar vs entry, p = 0.65 Familiar vs Novel, p = 0.99 Entry vs Novel, p = 0.44 |
|  |  |  |  |  |  | Entry | 87.69 | 11.97 |  |  |  |  |
|  |  |  |  |  |  | Novel | 119.00 | 12.69 |  |  |  |  |
| 2H |  | WT = 12 Chd2^+/-^ = 10 | Discrimination index | | WT | | 34.14 | 6.84 | Two-tailed  t-test | t(20) = 2.643,  p = 0.0156, d = 1.13 |  |  |
|  |  |  |  |  | Chd2^+/-^ | | -1.28 | 12.21 |  |  |  |  |
| 2I |  | Control = 6 Chd2^+/cKO^ = 6 | Time in arm (s) | | Control | Familiar | 70.05 | 9.89 | Two-way  ANOVA | Arm, F (2, 36) = 7.773, p = 0.0016, η²_p_ = 0.302 Genotype, F (1, 60) = 4.302e-007, p = 0.97,  η²_p_ = 3.49E-05 Interaction, F (2, 36) = 8.501, p = 0.00095, η²_p_ = 0.32 | Tukey | Familiar vs entry = 0.99 Familiar vs Novel = 0.00046 Entry vs Novel = 0.00055 |
|  |  |  |  |  |  | Entry | 77.22 | 8.65 |  |  |  |  |
|  |  |  |  |  |  | Novel | 154.38 | 9.58 |  |  |  |  |
|  |  |  |  |  | Chd2^+/cKO^ | Familiar | 73.07 | 24.74 |  |  | Tukey | Familiar vs entry = 0.14 Familiar vs Novel = 0.87 Entry vs Novel = 0.73 |
|  |  |  |  |  |  | Entry | 128.97 | 18.82 |  |  |  |  |
|  |  |  |  |  |  | Novel | 97.93 | 13.72 |  |  |  |  |
| 2J |  | Control = 6 Chd2^+/cKO^ = 6 | Discrimination index | | Control | | 33.37 | 6.89 | Two-tailed  t-test | t(10) = 4.53,  p = 0.00116, d = 2.61 |  |  |
|  |  |  |  |  | Chd2^+/cKO^ | | -13.64 | 7.75 |  |  |  |  |
| 2L | OLM male | WT = 9 Chd2^+/-^ = 13 | Discrimination index | | WT | Expose | 1.07 | 3.03 | Two-way  RM ANOVA | Phase, F (1, 20) = 56.39, p = 3.06E-07, η²_p_ = 0.76 Genotype, F (1, 20) = 34.24, p = 1.00E-05, η²_p_ = 0.74 Interaction, F (1, 20) = 58.39, p = 2.35E-07, η²_p_ = 0.74 | Tukey | WT, Expose vs Test, p = 4.05E-09 Chd2^+/-^, Expose vs Test, p = 0.92 Expose, WT vs Chd2^+/-^, p = 0.82 Test, WT vs Chd2^+/-^, p = 1.85E-11 |
|  |  |  |  |  |  | Test | 35.27 | 2.83 |  |  |  |  |
|  |  |  |  |  | Chd2^+/-^ | Expose | 0.21 | 2.73 |  |  |  |  |
|  |  |  |  |  |  | Test | -0.09 | 2.16 |  |  |  |  |
| 2M |  | Control = 12 Chd2^+/cKO^ = 10 | Discrimination index | | Control | Expose | 7.34 | 2.13 | Two-way  RM ANOVA | Phase, F (1, 20) = 39.62, p = 3.81E-06, η²_p_ = 0.66 Genotype, F (1, 20) = 31.85, p = 1.60E-05, η²_p_ = 0.68 Interaction, F (1, 20) = 18.53, p = 3.45E-04, η²_p_ = 0.48 | Tukey | Ctrl, Expose vs Test, p = 1.53E-07 Chd2^+/cKO^, Expose vs Test, p = 0.19 Expose, WT vs Chd2^+/cKO^, p = 0.16 Test, WT vs Chd2^+/cKO^, p = 1.44E-08 |
|  |  |  |  |  |  | Test | 48.79 | 6.43 |  |  |  |  |
|  |  |  |  |  | Chd2^+/cKO^ | Expose | -1.10 | 2.42 |  |  |  |  |
|  |  |  |  |  |  | Test | 6.68 | 3.54 |  |  |  |  |
| 2N | OLM female | WT =12 Chd2^+/-^ = 10 | Discrimination index | | WT | Expose | 1.13 | 1.81 | Two-way  RM ANOVA | Phase, F (1, 20) = 63.85, p = 1.19E-07, η²_p_ = 0.76 Genotype, F (1, 20) = 60.55, p = 1.78E-07, η²_p_ = 0.78 Interaction, F (1, 20) = 59.63, p = 2.00E-07, η²_p_ = 0.75 | Tukey | WT, Expose vs Test, p = 2.28E-10 Chd2^+/-^, Expose vs Test, p = 0.86 Expose, WT vs Chd2^+/-^, p = 0.69 Test, WT vs Chd2^+/-^, p = 1.29E-13 |
|  |  |  |  |  |  | Test | 42.13 | 3.44 |  |  |  |  |
|  |  |  |  |  | Chd2^+/-^ | Expose | -0.38 | 2.92 |  |  |  |  |
|  |  |  |  |  |  | Test | 0.32 | 2.13 |  |  |  |  |
| 2O |  | Control = 6 Chd2^+/cKO^ = 6 | Discrimination index | | Control | Expose | -1.41 | 3.77 | Two-way  RM ANOVA | Phase, F (1, 10) = 71.96, p = 7.02E-06, η²_p_ = 0.88 Genotype, F (1, 10) = 44.39, p = 5.62E-05, η²_p_ = 0.82 Interaction, F (1, 10) = 75.20, p = 5.77E-06, η²_p_ = 0.88 | Tukey | Ctrl, Expose vs Test, p = 2.64E-07 Chd2^+/cKO^, Expose vs Test, p = 0.9 Expose, WT vs Chd2^+/cKO^, p = 0.21 Test, WT vs Chd2^+/cKO^, p = 8.19E-10 |
|  |  |  |  |  |  | Test | 47.09 | 2.69 |  |  |  |  |
|  |  |  |  |  | Chd2^+/cKO^ | Expose | 3.85 | 2.53 |  |  |  |  |
|  |  |  |  |  |  | Test | 3.32 | 2.21 |  |  |  |  |
| 2Q | ORM male | WT = 9 Chd2^+/-^ = 13 | Discrimination index | | WT | Expose | -0.25 | 1.67 | Two-way  RM ANOVA | Phase, F (1, 20) = 64.19, p = 1.14E-07, η²_p_ = 0.76 Genotype, F (1, 20) = 55.35, p = 3.51E-07, η²_p_ = 0.67 Interaction, F (1, 20) = 42.68, p = 2.29E-06, η²_p_ = 0.68 | Tukey | WT, Expose vs Test, p = 7.97E-09 Chd2^+/-^, Expose vs Test, p = 0.26 Expose, WT vs Chd2^+/-^, p = 0.92 Test, WT vs Chd2^+/-^, p = 3.40E-12 |
|  |  |  |  |  |  | Test | 55.14 | 6.45 |  |  |  |  |
|  |  |  |  |  | Chd2^+/-^ | Expose | 0.23 | 1.86 |  |  |  |  |
|  |  |  |  |  |  | Test | 5.86 | 3.24 |  |  |  |  |
| 2R |  | Control = 12 Chd2^+/cKO^ = 10 | Discrimination index | | Control | Expose | 8.62 | 6.46 | Two-way  RM ANOVA | Phase, F (1, 20) = 142.7, p = 1.47E-10, η²_p_ = 0.88 Genotype, F (1, 20) = 6.983, p = 0.016, η²_p_ = 0.3 Interaction, F (1, 20) = 0.005553, p = 0.94,  η²_p_ = 0.00028 | Tukey | Ctrl, Expose vs Test, p = 2.58E-08 Chd2^+/cKO^, Expose vs Test, p = 0.00895 Expose, WT vs Chd2^+/cKO^, p = 0.051 Test, WT vs Chd2^+/cKO^, p = 0.064 |
|  |  |  |  |  |  | Test | 58.08 | 2.54 |  |  |  |  |
|  |  |  |  |  | Chd2^+/cKO^ | Expose | -3.83 | 3.94 |  |  |  |  |
|  |  |  |  |  |  | Test | 46.25 | 2.64 |  |  |  |  |
| 2S | ORM female | WT = 12 Chd2^+/-^ = 10 | Discrimination index | | WT | Expose | -0.91 | 1.99 | Two-way  RM ANOVA | Phase, F (1, 20) = 70.15, p = 5.70E-08, η²_p_ = 0.78 Genotype, F (1, 20) = 49.39, p = 8.12E-07, η²_p_ = 0.70 Interaction, F (1, 20) = 46.88, p = 1.18E-06, η²_p_ = 0.70 | Tukey | WT, Expose vs Test, p = 3.97E-10 Chd2^+/-^, Expose vs Test, p = 0.31 Expose, WT vs Chd2^+/-^, p = 0.97 Test, WT vs Chd2^+/-^, p = 3.37E-12 |
|  |  |  |  |  |  | Test | 47.86 | 3.29 |  |  |  |  |
|  |  |  |  |  | Chd2^+/-^ | Expose | -0.76 | 1.67 |  |  |  |  |
|  |  |  |  |  |  | Test | 4.14 | 4.87 |  |  |  |  |
| 2T |  | Control = 6 Chd2^+/cKO^ = 6 | Discrimination index | | Control | Expose | 0.50 | 1.24 | Two-way  RM ANOVA | Phase, F (1, 10) = 369.7, p = 3.15E-09, η²_p_ = 0.97 Genotype, F (1, 10) = 0.4249, p = 0.53, η²_p_ = 0.05 Interaction, F (1, 10) = 0.07554, p = 0.79, η²_p_ = 0.0075 | Tukey | Ctrl, Expose vs Test, p = 1.03E-07 Chd2^+/cKO^, Expose vs Test, p = 7.82E-08 Expose, WT vs Chd2^+/cKO^, p = 0.51 Test, WT vs Chd2^+/cKO^, p = 0.77 |
|  |  |  |  |  |  | Test | 45.89 | 3.72 |  |  |  |  |
|  |  |  |  |  | Chd2^+/cKO^ | Expose | -1.89 | 1.44 |  |  |  |  |
|  |  |  |  |  |  | Test | 44.82 | 2.84 |  |  |  |  |
| 2V | FC male | WT = 9 Chd2^+/-^ = 13 | Freezing | Context A | WT | | 44.25 | 1.60 | Two-way  RM ANOVA | Phase, F (1.440,28.80) = 99.71, p = 2.51E-12, η²_p_ = 0.83 Genotype, F (1, 20) = 15.73, p = 7.62E-04, η²_p_ = 0.20 Interaction, F (1.440,28.80) = 14.04, p = 2.19E-04, η²_p_ = 0.41 | Tukey | Context A, WT vs Chd2^+/-^, p = 2.06E-05 Context B, WT vs Chd2^+/-^, p = 0.17 Context B+cue, WT vs Chd2^+/-^, p = 0.4 WT, Context A vs Context B, p = 9.14E-09 Chd2^+/-^, Context A vs Context B, p = 0..83 |
|  |  |  |  |  | Chd2^+/-^ | | 20.89 | 3.74 |  |  |  |  |
|  |  |  |  | Context B  Context | WT | | 16.99 | 0.89 |  |  |  |  |
|  |  |  |  |  | Chd2^+/-^ | | 14.53 | 1.53 |  |  |  |  |
|  |  |  |  | Context B  Cue | WT | | 55.65 | 1.79 |  |  |  |  |
|  |  |  |  |  | Chd2^+/-^ | | 51.73 | 2.03 |  |  |  |  |
| 2W |  | Control = 12 Chd2^+/cKO^ = 10 | Freezing | Context A | Control | | 43.53 | 3.32 | Two-way  RM ANOVA | Phase, F (1.684, 33.68) = 151.9, p < 1.0E-15, η²_p_ = 0.88 Genotype, F (1, 20) = 11.26, p = 0.0031, η²_p_ = 0.44 Interaction, F (1.684, 33.6) = 19.54, p = 6.550E-06, η²_p_ = 0.49 | Tukey | Context A, WT vs Chd2^+/-^, p = 1.69E-04 Context B, WT vs Chd2^+/-^, p = 0.35 Context B+cue, WT vs Chd2^+/-^, p = 0.58 WT, Context A vs Context B, p = 1.65E-06 Chd2^+/-^, Context A vs Context B, p = 0.89 |
|  |  |  |  |  | Chd2^+/cKO^ | | 14.28 | 5.01 |  |  |  |  |
|  |  |  |  | Context B  Context | Control | | 14.32 | 1.67 |  |  |  |  |
|  |  |  |  |  | Chd2^+/cKO^ | | 12.25 | 1.37 |  |  |  |  |
|  |  |  |  | Context B  Cue | Control | | 57.41 | 2.71 |  |  |  |  |
|  |  |  |  |  | Chd2^+/cKO^ | | 54.84 | 3.63 |  |  |  |  |
| 2X | FC female | WT = 12 Chd2^+/-^ = 10 | Freezing | Context A | WT | | 45.35 | 2.08 | Two-way  RM ANOVA | Phase, F (1.641, 32.81) = 137.3, p = 1.0E-15, η²_p_ = 0.87 Genotype, F (1, 20) = 22.26, p = 0.00013, η²_p_ = 0.32 Interaction, F (1.641, 32.81) = 17.37, p = 2.11E-05, η²_p_ = 0.47 | Tukey | Context A, WT vs Chd2^+/-^, p = 1.94E-05 Context B, WT vs Chd2^+/-^, p = 0..32 Context B+cue, WT vs Chd2^+/-^, p = 0.84 WT, Context A vs Context B, p = 8.55E-11 Chd2^+/-^, Context A vs Context B, p = 0.02 |
|  |  |  |  |  | Chd2^+/-^ | | 20.24 | 3.38 |  |  |  |  |
|  |  |  |  | Context B  Context | WT | | 8.29 | 1.73 |  |  |  |  |
|  |  |  |  |  | Chd2^+/-^ | | 6.56 | 1.96 |  |  |  |  |
|  |  |  |  | Context B  Cue | WT | | 46.70 | 6.07 |  |  |  |  |
|  |  |  |  |  | Chd2^+/-^ | | 47.57 | 2.22 |  |  |  |  |
| 2Y |  | Control = 6 Chd2^+/cKO^ = 6 | Freezing | Context A | Control | | 43.05 | 3.24 | Two-way  RM ANOVA | Phase, F (1.837, 18.37) = 94.59, p = 3.42E-10, η²_p_ = 0.904 Genotype, F (1, 10) = 7.013, p = 0.024, η²_p_ = 0.36 Interaction, F (1.837, 18.37) = 13.60, p = 3.08E-04, η²_p_ = 0.58 | Tukey | Context A, WT vs Chd2^+/-^, p = 3.79E-04 Context B, WT vs Chd2^+/-^, p = 0.59 Context B+cue, WT vs Chd2^+/-^, p = 0.94 WT, Context A vs Context B, p = 4.00E-06 Chd2^+/-^, Context A vs Context B, p = 0.43 |
|  |  |  |  |  | Chd2^+/cKO^ | | 15.80 | 3.95 |  |  |  |  |
|  |  |  |  | Context B  Context | Control | | 6.84 | 3.08 |  |  |  |  |
|  |  |  |  |  | Chd2^+/cKO^ | | 8.85 | 1.83 |  |  |  |  |
|  |  |  |  | Context B  Cue | Control | | 50.87 | 5.20 |  |  |  |  |
|  |  |  |  |  | Chd2^+/cKO^ | | 50.44 | 2.01 |  |  |  |  |
| 3B | Sociability male | WT = 9 Chd2^+/-^ = 13 | Interaction time (s) | | WT | O1 | 79.89 | 5.00 | Two-way ANOVA | Genotype, F (1, 40) = 1.240, p = 0.27, η²_p_ = 0.03 Stimulus, F (1, 40) = 81.45, p = 3.41E-11, η²_p_ = 0.67 Interaction, F (1, 40) = 8.135, p = 0.0068, η²_p_ = 0.17 | Tukey | WT, Object vs Stranger 1, p = 1.11E-08 Chd2^+/-^, Object vs Stranger 1, p = 0.0118 |
|  |  |  |  |  |  | S1 | 157.89 | 10.22 |  |  |  |  |
|  |  |  |  |  | Chd2^+/-^ | O1 | 91.31 | 3.01 |  |  |  |  |
|  |  |  |  |  |  | S1 | 131.85 | 6.99 |  |  |  |  |
| 3C |  | WT = 9 Chd2^+/-^ = 13 | Social index | | WT | | 32.23 | 4.26 | Two-tailed  t-test | t(20) = 2.885, p = 0.0092, d = 1.23 |  |  |
|  |  |  |  |  | Chd2^+/-^ | | 17.51 | 3.06 |  |  |  |  |
| 3D |  | WT = 9 Chd2^+/-^ = 13 | Total obs. time (s) | | WT | | 237.78 | 10.64 | Two-tailed  t-test | t(20) = 1.188,  p = 0.2486, d = 0.51 |  |  |
|  |  |  |  |  | Chd2^+/-^ | | 223.15 | 7.14 |  |  |  |  |
| 3E |  | Control = 12 Chd2^+/cKO^ = 10 | Interaction time (s) | | Control | O1 | 64.67 | 7.38 | Two-way ANOVA | Genotype, F (1, 40) = 0.000228, p = 0.99,  η²_p_ = 5.70E-06 Stimulus, F (1, 40) = 91.48, p = 6.87E-12, η²_p_ = 0.7 Interaction, F(1,40) = 0.405, p = 0.53, η²_p_ = 0.01 | Tukey | Control, O1 vs S1, p = 3.73E-07 Chd2^+/cKO^, O1 vs S1, p = 1.50E-07 |
|  |  |  |  |  |  | S1 | 153.33 | 11.00 |  |  |  |  |
|  |  |  |  |  | Chd2^+/cKO^ | O1 | 58.20 | 10.08 |  |  |  |  |
|  |  |  |  |  |  | S1 | 159.50 | 10.92 |  |  |  |  |
| 3F |  | Control = 12 Chd2^+/cKO^ = 10 | Social index | | Control | | 42.00 | 3.43 | Two-tailed  t-test | t(20) = 0.77,  p = 0.452, d = 0.33 |  |  |
|  |  |  |  |  | Chd2^+/cKO^ | | 47.63 | 6.94 |  |  |  |  |
| 3G |  | Control = 12 Chd2^+/cKO^ = 10 | Total obs. time (s) | | Control | | 218.00 | 16.98 | Two-tailed  t-test | t(20) = 0.014, p = 0.989, d = 0.0063 |  |  |
|  |  |  |  |  | Chd2^+/cKO^ | | 217.70 | 12.88 |  |  |  |  |
| 3I | Sociability female | WT = 12 Chd2^+/-^ = 10 | Interaction time (s) | | WT | O1 | 70.17 | 5.80 | Two-way ANOVA | Genotype, F (1, 40) = 0.02922, p = 0.87, η²_p_ = 0.00073 Stimulus, F (1, 40) = 33.31, p = 9.92E-07, η²_p_ = 0.45 Interaction, F (1, 40) = 29.31, p = 3.14E-06, η²_p_ = 0.42 | Tukey | WT, O1 vs S1, p = 1.88E-09 Chd2^+/-^, O1 vs S1, p = 0.99 |
|  |  |  |  |  |  | S1 | 170.50 | 8.91 |  |  |  |  |
|  |  |  |  |  | Chd2^+/-^ | O1 | 117.20 | 9.89 |  |  |  |  |
|  |  |  |  |  |  | S1 | 120.40 | 11.24 |  |  |  |  |
| 3J |  | WT = 12 Chd2^+/-^ = 10 | Social index | | WT | | 41.54 | 4.00 | Two-tailed  t-test | t(20) = 8.833,  p = 2.44E-08, d = 3.79 |  |  |
|  |  |  |  |  | Chd2^+/-^ | | 1.05 | 1.44 |  |  |  |  |
| 3K |  | WT = 12 Chd2^+/-^ = 10 | Total obs. time (s) | | WT | | 240.67 | 10.26 | Two-tailed  t-test | t(20) = 0.1390,  p = 0.89, d = 0.059 |  |  |
|  |  |  |  |  | Chd2^+/-^ | | 237.60 | 20.88 |  |  |  |  |
| 3L |  | Control = 6 Chd2^+/cKO^ = 6 | Interaction time (s) | | Control | O1 | 70.00 | 7.94 | Two-way ANOVA | Genotype, F(1,20) = 0.0578, p = 0.81, η²_p_ = 0.0029 Stimulus, F(1,20) = 119.2, p = 7.08E-10, η²_p_ = 0.86 Interaction, F(1,20) = 0.57, p = 0.46, η²_p_ = 0.027 | Tukey | Control, O1 vs S1, p = 4.08E-07 Chd2^+/cKO^, O1 vs S1, p = 3.29E-06 |
|  |  |  |  |  |  | S1 | 191.33 | 13.30 |  |  |  |  |
|  |  |  |  |  | Chd2^+/cKO^ | O1 | 75.33 | 8.07 |  |  |  |  |
|  |  |  |  |  |  | S1 | 181.00 | 11.28 |  |  |  |  |
| 3M |  | Control = 6 Chd2^+/cKO^ = 6 | Social index | | Control | | 46.15 | 5.86 | Two-tailed  t-test | t(10) = 0.56,  p = 0.59, d = 0.32 |  |  |
|  |  |  |  |  | Chd2^+/cKO^ | | 41.22 | 5.44 |  |  |  |  |
| 3N |  | Control = 6 Chd2^+/cKO^ = 6 | Total obs. time (s) | | Control | | 261.33 | 11.80 | Two-tailed  t-test | t(10) = 0.28,  p = 0.78, d = 0.16 |  |  |
|  |  |  |  |  | Chd2^+/cKO^ | | 256.33 | 13.17 |  |  |  |  |
| 3P | Social  novelty male | WT = 9 Chd2^+/-^ = 13 | Interaction time (s) | | WT | S1 | 64.89 | 9.93 | Two-way ANOVA | Genotype, F (1, 40) = 11.63, p = 0.0015, η²_p_ = 0.005 Stimulus, F (1, 40) = 12.99, p = 0.00086, η²_p_ = 0.25 Interaction, F (1, 40) = 11.63, p = 0.0015, η²_p_ = 0.23 | Tukey | WT, S1 vs S2, p = 2.67E-04 Chd2^+/-^, S1 vs S2, p = 0.99 |
|  |  |  |  |  |  | S2 | 156.44 | 14.07 |  |  |  |  |
|  |  |  |  |  | Chd2^+/-^ | S1 | 103.08 | 13.42 |  |  |  |  |
|  |  |  |  |  |  | S2 | 105.62 | 12.15 |  |  |  |  |
| 3Q |  | WT = 9 Chd2^+/-^ = 13 | Social index | | WT | | 42.74 | 6.04 | Two-tailed  t-test | t(20) = 3.500,  p = 0.0023, d = 1.49 |  |  |
|  |  |  |  |  | Chd2^+/-^ | | 2.29 | 8.62 |  |  |  |  |
| 3R |  | WT = 9 Chd2^+/-^ = 13 | Total obs. time (s) | | WT | | 221.33 | 19.77 | Two-tailed  t-test | t(20) = 0.5376,  p = 0.4038, d = 0.23 |  |  |
|  |  |  |  |  | Chd2^+/-^ | | 208.69 | 14.02 |  |  |  |  |
| 3S |  | Control = 12 Chd2^+/cKO^ = 10 | Interaction time (s) | | Control | S1 | 81.33 | 5.70 | Two-way ANOVA | Genotype, F(1,40) = 0.6960, p = 0.41, η²_p_ = 0.02 Stimulus, F(1,40) = 144.4, p = 8E-15, η²_p_ = 0.78 Interaction, F(1,40) = 2.507, p = 0.12, η²_p_ = 0.06 | Tukey | Control, S1 vs S2, p = 9.52E-12 Chd2^+/cKO^, S1 vs S2, p= 9.07E-08 |
|  |  |  |  |  |  | S2 | 187.83 | 11.82 |  |  |  |  |
|  |  |  |  |  | Chd2^+/cKO^ | S1 | 87.20 | 4.09 |  |  |  |  |
|  |  |  |  |  |  | S2 | 168.90 | 5.31 |  |  |  |  |
| 3T |  | Control = 12 Chd2^+/cKO^ = 10 | Social index | | Control | | 39.43 | 2.40 | Two-tailed  t-test | t(20) = 1.236,  p = 0.0692, d = 0.53 |  |  |
|  |  |  |  |  | Chd2^+/cKO^ | | 32.09 | 2.16 |  |  |  |  |
| 3U |  | Control = 12 Chd2^+/cKO^ = 10 | Total obs. time (s) | | Control | | 269.17 | 16.26 | Two-tailed  t-test | t(20) = 0.6782,  p = 0.5054, d = 0.29 |  |  |
|  |  |  |  |  | Chd2^+/cKO^ | | 256.10 | 7.89 |  |  |  |  |
| 3W | Social  novelty female | WT = 12 Chd2^+/-^ = 10 | Interaction time (s) | | WT | S1 | 57.83 | 3.98 | Two-way ANOVA | Genotype, F (1, 40) = 0.2121, p = 0.65, η²_p_ = 0.0053 time, F (1, 40) = 31.35, p = 1.73E-06, η²_p_ = 0.44 Interaction,F (1, 40) = 56.10, p = 3.93E-09, η²_p_ = 0.58 | Tukey | WT, S1 vs S2, p = 2.74E-11 Chd2^+/-^, S1 vs S2, p= 0.58 |
|  |  |  |  |  |  | S2 | 129.83 | 3.47 |  |  |  |  |
|  |  |  |  |  | Chd2^+/-^ | S1 | 96.50 | 7.24 |  |  |  |  |
|  |  |  |  |  |  | S2 | 86.10 | 7.31 |  |  |  |  |
| 3X |  | WT = 12 Chd2^+/-^ = 10 | Social index | | WT | | 38.98 | 3.30 | Two-tailed  t-test | t(20) = 5.688,  p = 1.44E-05, d = 2.43 |  |  |
|  |  |  |  |  | Chd2^+/-^ | | -5.72 | 7.67 |  |  |  |  |
| 3Y |  | WT = 12 Chd2^+/-^ = 10 | Total obs. time (s) | | WT | | 187.67 | 5.66 | Two-tailed  t-test | t(20) = 0.6594,  p = 0.5172, d = 0.28 |  |  |
|  |  |  |  |  | Chd2^+/-^ | | 182.60 | 4.96 |  |  |  |  |
| 3Z |  | Control = 6 Chd2^+/cKO^ = 6 | Interaction time (s) | | Control | S1 | 44.33 | 9.49 | Two-way ANOVA | Genotype, F(1,20) = 0.0146, p = 0.91, η²_p_ = 0.00073 Time, F(1,20) = 189.6, p = 1.16E-11, η²_p_ = 0.905 Interaction, F(1,20) = 0.027, p = 0.87, η²_p_ = 0.0014 | Tukey | Control, S1 vs S2, p = 2.33E-08 Chd2^+/cKO^, S1 vs S2, p = 3.46E-08 |
|  |  |  |  |  |  | S2 | 150.17 | 8.85 |  |  |  |  |
|  |  |  |  |  | Chd2^+/cKO^ | S1 | 44.67 | 4.68 |  |  |  |  |
|  |  |  |  |  |  | S2 | 148.00 | 6.37 |  |  |  |  |
| 3AA |  | Control = 6 Chd2^+/cKO^ = 6 | Social index | | Control | | 54.75 | 9.05 | Two-tailed  t-test | t(10) = 0.11,  p = 0.917, d = 0.06 |  |  |
|  |  |  |  |  | Chd2^+/cKO^ | | 53.67 | 4.60 |  |  |  |  |
| 3BB |  | Control = 6 Chd2^+/cKO^ = 6 | Total obs. time (s) | | Control | | 194.50 | 5.08 | Two-tailed  t-test | t(10) = 0.23,  p = 0.826, d = 0.13 |  |  |
|  |  |  |  |  | Chd2^+/cKO^ | | 192.67 | 6.35 |  |  |  |  |
| 4C | aSD male | WT = 6 Chd2^+/-^ = 6 | Defeat | Time in  Far zone (s) | WT | | 171.10 | 5.60 | Two-way ANOVA | Genotype, F (1, 20) = 3.776, p = 0.066, η²_p_ = 0.66 Zone, F (1, 20) = 682.3, p = 1.74E-13, η²_p_ = 0.97 Interaction, F (1, 20) = 41.08, p = 2.98E-06, η²_p_ = 0.67 | Tukey | Far zone  WT vs Chd2^+/-^, p = 0.0049  Interactive zone  WT vs Chd2^+/-^, p = 0.0024 |
|  |  |  |  |  | Chd2^+/-^ | | 225.62 | 8.85 |  |  |  |  |
|  |  |  |  | Time in  interactive zone (s) | WT | | 42.45 | 6.36 |  |  |  |  |
|  |  |  |  |  | Chd2^+/-^ | | 13.30 | 4.49 |  |  |  |  |
| 4D |  | WT = 6 Chd2^+/-^ = 6 | No Defeat | Time in  Far zone (s) | WT | | 70.48 | 8.49 | Two-way ANOVA | Genotype, F (1, 20) = 0.01004, p = 0.92, η²_p_ = 0.29 Zone, F (1, 20) = 28.21, p = 3.37E-05, η²_p_ = 0.59 Interaction, F (1, 20) = 8.028, p = 0.0102, η²_p_ = 0.0005 | Tukey | Far zone  WT vs Chd2^+/-^, p = 0.25  Interactive zone  WT vs Chd2^+/-^, p = 0.2 |
|  |  |  |  |  | Chd2^+/-^ | | 89.97 | 1.62 |  |  |  |  |
|  |  |  |  | Time in  interactive zone (s) | WT | | 128.55 | 9.89 |  |  |  |  |
|  |  |  |  |  | Chd2^+/-^ | | 107.63 | 5.55 |  |  |  |  |
| 4E |  | Control = 7 Chd2^+/cKO^ = 6 | Defeat | Time in  Far zone (s) | Control | | 163.37 | 11.02 | Two-way ANOVA | Genotype, F (1, 22) = 3.372, p = 0.08, η²_p_ = 0.13 Zone, F (1, 22) = 305.9, p = 2.2E-14, η²_p_ = 0.93 Interaction, F (1, 22) = 15.14, p = 7.87E-04, η²_p_ = 0.41 | Tukey | Far zone  WT vs Chd2^+/cKO^, p = 0.0028  Interactive zone  WT vs Chd2^+/cKO^, p = 0.048 |
|  |  |  |  |  | Chd2^+/cKO^ | | 218.58 | 14.50 |  |  |  |  |
|  |  |  |  | Time in  interactive zone (s) | Control | | 33.68 | 6.63 |  |  |  |  |
|  |  |  |  |  | Chd2^+/cKO^ | | 12.45 | 2.28 |  |  |  |  |
| 4F |  | Control = 6 Chd2^+/cKO^ = 5 | No Defeat | Time in  Far zone (s) | Control | | 78.63 | 19.68 | Two-way ANOVA | Genotype, F (1, 18) = 0.06155, p = 0.81, η²_p_ = 0.0034 Zone, F (1, 18) = 5.993, p = 0.025, η²_p_ = 0.25 Interaction, F (1, 18) = 1.461e-005, p = 0.99,  η²_p_ = 8.12E-07 | Tukey | Far zone  WT vs Chd2^+/cKO^, p = 0.99  Interactive zone  WT vs Chd2^+/cKO^, p = 0.99 |
|  |  |  |  |  | Chd2^+/cKO^ | | 74.16 | 16.36 |  |  |  |  |
|  |  |  |  | Time in  interactive zone (s) | Control | | 123.53 | 17.94 |  |  |  |  |
|  |  |  |  |  | Chd2^+/cKO^ | | 118.92 | 17.74 |  |  |  |  |
| 4G | aSD female | WT = 7 Chd2^+/-^ = 8 | Defeat | Time in  Far zone (s) | WT | | 171.97 | 9.42 | Two-way ANOVA | Genotype, F (1, 26) = 0.0003134, p = 0.99,  η²_p_ = 1.21E-05 Zone, F (1, 26) = 265.1, p = 4E-15, η²_p_ = 0.91 Interaction, F (1, 26) = 0.03223, p = 0.86, η²_p_ = 0.0012 | Tukey | Far zone  WT vs Chd2^+/-^, p = 0.99  Interactive zone  WT vs Chd2^+/-^, p = 0.99 |
|  |  |  |  |  | Chd2^+/-^ | | 170.35 | 10.36 |  |  |  |  |
|  |  |  |  | Time in  interactive zone (s) | WT | | 36.66 | 5.48 |  |  |  |  |
|  |  |  |  |  | Chd2^+/-^ | | 37.99 | 6.15 |  |  |  |  |
| 4H |  | WT = 7 Chd2^+/-^ = 7 | No Defeat | Time in  Far zone (s) | WT | | 86.84 | 10.28 | Two-way ANOVA | Genotype, F (1, 24) = 1.065, p = 0.31, η²_p_ = 0.042 Zone, F (1, 24) = 31.73, p = 8.46E-06, η²_p_ = 0.57 Interaction, F (1, 24) = 0.1683, p = 0.69, η²_p_ = 0.007 | Tukey | Far zone  WT vs Chd2^+/-^, p = 0.97  Interactive zone  WT vs Chd2^+/-^, p = 0.74 |
|  |  |  |  |  | Chd2^+/-^ | | 82.46 | 7.21 |  |  |  |  |
|  |  |  |  | Time in  interactive zone (s) | WT | | 129.46 | 4.00 |  |  |  |  |
|  |  |  |  |  | Chd2^+/-^ | | 119.29 | 5.03 |  |  |  |  |
| 4I |  | Control = 8 Chd2^+/cKO^ = 7 | Defeat | Time in  Far zone (s) | Control | | 189.90 | 8.80 | Two-way ANOVA | Genotype, F (1, 26) = 0.7090, p = 0.41, η²_p_ = 0.027 Zone, F (1, 26) = 585.8, p = 9.56E-14, η²_p_ = 0.96 Interaction, F (1, 26) = 0.09102, p = 0.77, η²_p_ = 0.0035 | Tukey | Far zone  WT vs Chd2^+/-^, p= 0.98  Interactive zone  WT vs Chd2^+/-^, p = 0.85 |
|  |  |  |  |  | Chd2^+/cKO^ | | 186.44 | 3.86 |  |  |  |  |
|  |  |  |  | Time in  interactive zone (s) | Control | | 36.98 | 5.44 |  |  |  |  |
|  |  |  |  |  | Chd2^+/cKO^ | | 29.66 | 5.62 |  |  |  |  |
| 4J |  | WT = 7 Chd2^+/cKO^ = 7 | No Defeat | Time in  Far zone (s) | Control | | 103.86 | 10.41 | Two-way ANOVA | Genotype, F (1, 24) = 2.062, p = 0.16, η²_p_ = 0.079 Zone, F (1, 24) = 5.544, p = 0.027, η²_p_ = 0.19 Interaction, F (1, 24) = 0.8817, p = 0.36, η²_p_ = 0.035 | Tukey | Far zone  WT vs Chd2^+/-^, p = 0.36  Interactive zone  WT vs Chd2^+/-^, p = 0.98 |
|  |  |  |  |  | Chd2^+/cKO^ | | 87.13 | 4.18 |  |  |  |  |
|  |  |  |  | Time in  interactive zone (s) | Control | | 113.83 | 7.57 |  |  |  |  |
|  |  |  |  |  | Chd2^+/cKO^ | | 110.33 | 3.92 |  |  |  |  |
| 6F | Sociability male | Vehicle  WT = 8  Chd2^+/-^ = 6  L-838,417  WT = 8 Chd2^+/-^ = 6 | Interaction  time (s) | Vehicle | WT | O1 | 60.63 | 2.85 | Two-way  RM ANOVA | Stimulus, F (1, 24) = 424.8, p = 1.25E-15, η²_p_ = 0.94 Treatment, F (1, 24) = 0.0009533, p = 0.98,  η²_p_ = 3.97E-05 Genotype, F (1, 24) = 0.08090, p = 0.78, η²_p_ = 0.0030 Stimulus x Treatment, F (1, 24) = 20.34, p = 0.00014, η²_p_ = 0.46 Stimulus, Genotype, F (1, 24) = 17.14, p = 0.00037,  η²_p_ = 0.39 Treatment x Genotype, F (1, 24) = 0.07460, p = 0.79, η²_p_ = 0.0031 Stimulus x Treatment x Genotype, F (1, 24) = 10.51,  p = 0.0035, η²_p_ = 0.305 | Tukey | Vehicle, WT, O1 vs S1, p = 1.06E-13 Vehicle, Chd2^+/-^, O1 vs S1, p = 0.0104 L-838,417, WT, O1 vs S1, p = 9.44E-14 L-838,417, Chd2^+/-^, O1 vs S1, p = 8.94E-13 |
|  |  |  |  |  |  | S1 | 157.50 | 6.75 |  |  |  |  |
|  |  |  |  |  | Chd2^+/-^ | O1 | 92.00 | 3.28 |  |  |  |  |
|  |  |  |  |  |  | S1 | 126.17 | 2.66 |  |  |  |  |
|  |  |  |  | L-838,417 | WT | O2 | 56.13 | 3.25 |  |  |  |  |
|  |  |  |  |  |  | S2 | 164.13 | 8.35 |  |  |  |  |
|  |  |  |  |  | Chd2^+/-^ | O2 | 56.67 | 5.51 |  |  |  |  |
|  |  |  |  |  |  | S2 | 158.83 | 10.03 |  |  |  |  |
| 6G |  | Vehicle  WT = 8  Chd2^+/-^ = 6  L-838,417  WT = 8  Chd2^+/-^ = 6 | Social index | Vehicle | WT | | 44.39 | 1.47 | Two-way  RM ANOVA | Genotype, F (1, 12) = 24.32, p = 0.00035, η²_p_ = 0.61 Treatment, F (1, 12) = 25.67, p = 0.00028, η²_p_ = 0.68 Interaction, F (1, 12) = 14.95, p = 0.0022, η²_p_ = 0.55 | Tukey | Vehicle, WT vs Chd2^+/-^, p = 2.3E-06 L-838,417, WT vs Chd2^+/-^, p = 0.73 |
|  |  |  |  |  | Chd2^+/-^ | | 15.73 | 1.86 |  |  |  |  |
|  |  |  |  | L-838,417 | WT | | 48.59 | 3.39 |  |  |  |  |
|  |  |  |  |  | Chd2^+/-^ | | 46.95 | 5.52 |  |  |  |  |
| 6I | Sociability female | Vehicle  WT = 6 Chd2^+/-^ = 7  L-838,417  WT = 6 Chd2^+/-^ = 7 | Interaction  time (s) | Vehicle | WT | O1 | 60.67 | 2.43 | Two-way  RM ANOVA | Stimulus, F (1, 22) = 368.3, p = 3E-15, η²_p_ = 0.95 Treatment, F (1, 22) = 0.02792, p = 0.87, η²_p_ = 0.0013 Genotype, F (1, 22) = 0.1871, p = 0.19, η²_p_ = 0.01 Stimulus x Treatment, F (1, 22) = 42.32, p = 1.52E-06, η²_p_ = 0.66 Stimulus, Genotype, F (1, 22) = 46.88, p = 7.06E-07, η²_p_ = 0.72 Treatment x Genotype, F (1, 22) = 0.1044, p = 0.75, η²_p_ = 0.0047 Stimulus x Treatment x Genotype, F (1, 22) = 75.49,  p = 1.46E-08, η²_p_ = 0.77 | Tukey | Vehicle, WT, O1 vs S1, p = 8.41E-13 Vehicle, Chd2^+/-^, O1 vs S1, p = 0.99 L-838,417, WT, O1 vs S1, p = 9E-13 L-838,417, Chd2^+/-^, O1 vs S1, p = 8.4E-13 |
|  |  |  |  |  |  | S1 | 158.67 | 4.99 |  |  |  |  |
|  |  |  |  |  | Chd2^+/-^ | O1 | 115.00 | 4.92 |  |  |  |  |
|  |  |  |  |  |  | S1 | 109.43 | 5.08 |  |  |  |  |
|  |  |  |  | L-838,417 | WT | O2 | 66.00 | 4.07 |  |  |  |  |
|  |  |  |  |  |  | S2 | 154.43 | 6.13 |  |  |  |  |
|  |  |  |  |  | Chd2^+/-^ | O2 | 66.67 | 4.99 |  |  |  |  |
|  |  |  |  |  |  | S2 | 154.67 | 4.09 |  |  |  |  |
| 6J |  | Vehicle  WT = 6 Chd2^+/-^ = 7  L-838,417  WT = 6 Chd2^+/-^ = 7 | Social index | Vehicle | WT | | 44.69 | 1.50 | Two-way  RM ANOVA | Genotype, F (1, 12) = 43.73, p = 9.44E-14, η²_p_ = 0.82 Treatment, F (1, 12) = 42.50, p = 8.78E-12, η²_p_ = 0.78 Interaction, F (1, 12) = 58.92, p = 8.47E-13, η²_p_ = 0.83 | Tukey | Vehicle, WT vs Chd2^+/-^, p = 9.75E-14 L-838,417, WT vs Chd2^+/-^, p = 0.92 |
|  |  |  |  |  | Chd2^+/-^ | | -2.52 | 3.06 |  |  |  |  |
|  |  |  |  | L-838,417 | WT | | 40.01 | 3.85 |  |  |  |  |
|  |  |  |  |  | Chd2^+/-^ | | 40.08 | 3.50 |  |  |  |  |
| S1A | Y-maze male | WT = 9 Chd2^+/-^ = 13 | Distance | | WT | | 7.31 | 0.68 | Two-tailed  t-test | t(20) = 0.43,  p = 0.6719, d = 0.18 |  |  |
|  |  |  |  |  | Chd2^+/-^ | | 6.95 | 0.53 |  |  |  |  |
| S1B |  | WT = 9 Chd2^+/-^ = 13 | Alternation | | WT | | 58.39 | 3.33 | Two-tailed  t-test | t(20) = 0.64, p = 0.5313, d = 0.27 |  |  |
|  |  |  |  |  | Chd2^+/-^ | | 60.76 | 2.06 |  |  |  |  |
| S1C |  | WT = 9 Chd2^+/-^ = 13 | Total arm entries | | WT | | 20.00 | 0.69 | Two-tailed  t-test | t(20) = 1.49,  p = 0.1513, d = 0.64 |  |  |
|  |  |  |  |  | Chd2^+/-^ | | 17.85 | 1.10 |  |  |  |  |
| S1D |  | Control = 12 Chd2^+/cKO^ = 10 | Distance | | Control | | 7.70 | 0.36 | Two-tailed  t-test | t(20) = 0.72,  p = 0.481, d = 0.31 |  |  |
|  |  |  |  |  | Chd2^+/cKO^ | | 7.06 | 0.86 |  |  |  |  |
| S1E |  | Control = 12 Chd2^+/cKO^ = 10 | Alternation | | Control | | 55.77 | 2.53 | Two-tailed  t-test | t(20) = 1.602,  p = 0.125, d = 0.68 |  |  |
|  |  |  |  |  | Chd2^+/cKO^ | | 62.12 | 3.10 |  |  |  |  |
| S1F |  | Control = 12 Chd2^+/cKO^ = 10 | Total arm entries | | Control | | 21.17 | 1.26 | Two-tailed  t-test | t(20) = 1.41,  p = 0.173, d = 0.60 |  |  |
|  |  |  |  |  | Chd2^+/cKO^ | | 18.50 | 1.41 |  |  |  |  |
| S1G | Y-maze female | WT = 12 Chd2^+/-^ = 10 | Distance | | WT | | 7.78 | 0.78 | Two-tailed  t-test | t(20) = 0.39, p = 0.6947, d = 0.17 |  |  |
|  |  |  |  |  | Chd2^+/-^ | | 8.17 | 0.48 |  |  |  |  |
| S1H |  | WT = 12 Chd2^+/-^ = 10 | Alternation | | WT | | 59.61 | 2.76 | Two-tailed  t-test | t(20) = 0.84,  p = 0.4107, d = 0.36 |  |  |
|  |  |  |  |  | Chd2^+/-^ | | 55.53 | 4.18 |  |  |  |  |
| S1I |  | WT = 12 Chd2^+/-^ = 10 | Total arm entries | | WT | | 16.67 | 2.14 | Two-tailed  t-test | t(20) = 0.13,  p = 0.8975, d = 0.055 |  |  |
|  |  |  |  |  | Chd2^+/-^ | | 17.00 | 1.11 |  |  |  |  |
| S1J |  | Control = 6 Chd2^+/cKO^ = 6 | Distance | | Control | | 8.66 | 0.76 | Two-tailed  t-test | t(10) = 0.44,  p = 0.668, d = 0.25 |  |  |
|  |  |  |  |  | Chd2^+/cKO^ | | 9.34 | 1.33 |  |  |  |  |
| S1K |  | Control = 6 Chd2^+/cKO^ = 6 | Alternation | | Control | | 62.14 | 2.93 | Two-tailed  t-test | t(10) = 2.16,  p = 0.0559, d = 1.25 |  |  |
|  |  |  |  |  | Chd2^+/cKO^ | | 53.19 | 2.72 |  |  |  |  |
| S1L |  | Control = 6 Chd2^+/cKO^ = 6 | Total arm entries | | Control | | 18.67 | 2.20 | Two-tailed  t-test | t(10) = 0.37, p = 0.716, d = 0.21 |  |  |
|  |  |  |  |  | Chd2^+/cKO^ | | 20.17 | 3.34 |  |  |  |  |
| S1M | OLM male | WT = 9 Chd2^+/-^ = 13 | Total obs. time (s) | | Expose | WT | 39.56 | 5.27 | Two-tailed  t-test | t(20) = 1.49,  p = 0.1501, d = 0.63 |  |  |
|  |  |  |  |  |  | Chd2^+/-^ | 31.62 | 2.52 |  |  |  |  |
| S1N |  | WT = 9 Chd2^+/-^ = 13 | Total obs. time (s) | | Test | WT | 23.22 | 1.48 | Two-tailed  t-test | t(20) = 1.56,  p = 0.1345, d = 0.67 |  |  |
|  |  |  |  |  |  | Chd2^+/-^ | 19.62 | 1.62 |  |  |  |  |
| S1O |  | Control = 12 Chd2^+/cKO^ = 10 | Total obs. time (s) | | Expose | Control | 46.08 | 7.23 | Two-tailed  t-test | t(20) = 0.73,  p = 0.475, d = 0.31 |  |  |
|  |  |  |  |  |  | Chd2^+/cKO^ | 39.10 | 5.92 |  |  |  |  |
| S1P |  | Control = 12 Chd2^+/cKO^ = 10 | Total obs. time (s) | | Test | Control | 31.25 | 4.31 | Two-tailed  t-test | t(20) = 1.24,  p = 0.231, d = 0.53 |  |  |
|  |  |  |  |  |  | Chd2^+/cKO^ | 24.30 | 3.75 |  |  |  |  |
| S1Q | OLM female | WT = 12 Chd2^+/-^ = 10 | Total obs. time (s) | | Expose | WT | 35.50 | 2.65 | Two-tailed  t-test | t(20) = 0.39,  p = 0.7015, d = 0.17 |  |  |
|  |  |  |  |  |  | Chd2^+/-^ | 37.20 | 3.59 |  |  |  |  |
| S1R |  | WT = 12 Chd2^+/-^ = 10 | Total obs. time (s) | | Test | WT | 18.92 | 1.26 | Two-tailed  t-test | t(20) = 1.72,  p = 0.1005, d = 0.73 |  |  |
|  |  |  |  |  |  | Chd2^+/-^ | 16.00 | 1.07 |  |  |  |  |
| S1S |  | Control = 6 Chd2^+/cKO^ = 6 | Total obs. time (s) | | Expose | Control | 31.33 | 9.30 | Two-tailed  t-test | t(10) = 0.104,  p = 0.919, d = 0.06 |  |  |
|  |  |  |  |  |  | Chd2^+/cKO^ | 32.33 | 2.26 |  |  |  |  |
| S1T |  | Control = 6 Chd2^+/cKO^ = 6 | Total obs. time (s) | | Test | Control | 18.83 | 0.70 | Two-tailed  t-test | t(10) = 1.48,  p = 0.169, d = 0.85 |  |  |
|  |  |  |  |  |  | Chd2^+/cKO^ | 16.50 | 1.41 |  |  |  |  |
| S1U | ORM male | WT = 9 Chd2^+/-^ = 13 | Total obs. time (s) | | Expose | WT | 34.00 | 2.13 | Two-tailed  t-test | t(20) = 0.02,  p = 0.9846, d = 0.0085 |  |  |
|  |  |  |  |  |  | Chd2^+/-^ | 33.92 | 2.91 |  |  |  |  |
| S1V |  | WT = 9 Chd2^+/-^ = 13 | Total obs. time (s) | | Test | WT | 23.67 | 3.53 | Two-tailed  t-test | t(20) = 0.07,  p = 0.9458, d = 0.03 |  |  |
|  |  |  |  |  |  | Chd2^+/-^ | 24.00 | 3.20 |  |  |  |  |
| S1W |  | Control = 12 Chd2^+/cKO^ = 10 | Total obs. time (s) | | Expose | Control | 34.33 | 1.74 | Two-tailed  t-test | t(20) = 0.33,  p = 0.74, d = 0.14 |  |  |
|  |  |  |  |  |  | Chd2^+/cKO^ | 35.60 | 3.70 |  |  |  |  |
| S1X |  | Control = 12 Chd2^+/cKO^ = 10 | Total obs. time (s) | | Test | Control | 27.83 | 4.87 | Two-tailed  t-test | t(20) = 0.0046,  p = 0.996, d = 0.002 |  |  |
|  |  |  |  |  |  | Chd2^+/cKO^ | 27.80 | 5.28 |  |  |  |  |
| S1Y | ORM female | WT = 7 Chd2^+/-^ = 7 | Total obs. time (s) | | Expose | WT | 29.33 | 1.49 | Two-tailed  t-test | t(20) = 0.9937,  p = 0.3323, d = 0.42 |  |  |
|  |  |  |  |  |  | Chd2^+/-^ | 31.80 | 2.05 |  |  |  |  |
| S1Z |  | WT = 7 Chd2^+/-^ = 7 | Total obs. time (s) | | Test | WT | 19.00 | 1.71 | Two-tailed  t-test | t(20) = 0.16,  p = 0.8705, d = 0.068 |  |  |
|  |  |  |  |  |  | Chd2^+/-^ | 18.60 | 1.68 |  |  |  |  |
| S1AA |  | Control = 6 Chd2^+/cKO^ = 6 | Total obs. time (s) | | Expose | Control | 33.83 | 1.94 | Two-tailed  t-test | t(10) = 1.89,  p = 0.0886, d = 1.09 |  |  |
|  |  |  |  |  |  | Chd2^+/cKO^ | 29.17 | 1.54 |  |  |  |  |
| S1BB |  | Control = 6 Chd2^+/cKO^ = 6 | Total obs. time (s) | | Test | Control | 15.17 | 1.76 | Two-tailed  t-test | t(10) = 0.5896,  p = 0.569, d = 0.34 |  |  |
|  |  |  |  |  |  | Chd2^+/cKO^ | 16.67 | 1.84 |  |  |  |  |
| S2A | aSD male | WT = 6 Chd2^+/-^ = 6 | Time defeated (s) | | WT | | 67.17 | 6.18 | Two-tailed  t-test | t(10) = 0.31,  p = 0.762, d = 0.18 |  |  |
|  |  |  |  |  | Chd2^+/-^ | | 69.83 | 5.91 |  |  |  |  |
| S2C |  | Control = 7 Chd2^+/cKO^ = 6 | Time defeated (s) | | Control | | 62.29 | 2.52 | Two-tailed  t-test | t(11) = 0.43,  p = 0.675, d = 0.24 |  |  |
|  |  |  |  |  | Chd2^+/cKO^ | | 64.83 | 5.70 |  |  |  |  |
| S2E | aSD female | WT = 7 Chd2^+/-^ = 8 | Time defeated (s) | | WT | | 54.57 | 1.70 | Two-tailed  t-test | t(13) = 0.8,  p = 0.4383, d = 0.41 |  |  |
|  |  |  |  |  | Chd2^+/-^ | | 52.50 | 1.91 |  |  |  |  |
| S2G |  | Control = 8 Chd2^+/cKO^ = 7 | Time defeated (s) | | Control | | 50.38 | 0.75 | Two-tailed  t-test | t(13) = 1.51,  p = 0.1551, d = 0.78 |  |  |
|  |  |  |  |  | Chd2^+/cKO^ | | 54.00 | 2.43 |  |  |  |  |
| S3B | OFT male | WT = 9 Chd2^+/-^ = 13 | Distance | | WT | | 20.25 | 0.94 | Two-tailed  t-test | t(20) = 0.66, p = 0.5163, d = 0.28 |  |  |
|  |  |  |  |  | Chd2^+/-^ | | 21.58 | 1.53 |  |  |  |  |
| S3C |  | WT = 9 Chd2^+/-^ = 13 | % time in center zone (s) | | WT | | 13.64 | 0.88 | Two-tailed  t-test | t(20) = 0.57, p = 0.5772, d = 0.24 |  |  |
|  |  |  |  |  | Chd2^+/-^ | | 11.94 | 2.39 |  |  |  |  |
| S3D | OFT female | WT = 12 Chd2^+/-^ = 10 | Distance | | WT | | 23.50 | 1.41 | Two-tailed  t-test | t(20) = 0.05,  p = 0.9599, d = 0.02 |  |  |
|  |  |  |  |  | Chd2^+/-^ | | 23.61 | 1.63 |  |  |  |  |
| S3E |  | WT = 12 Chd2^+/-^ = 10 | % time in center zone (s) | | WT | | 14.35 | 0.94 | Two-tailed  t-test | t(20) = 0.33,  p = 0.7461, d = 0.14 |  |  |
|  |  |  |  |  | Chd2^+/-^ | | 13.79 | 2.00 |  |  |  |  |
| S3G | OFT male | Control = 12 Chd2^+/cKO^ = 10 | Distance | | Control | | 20.82 | 1.41 | Two-tailed  t-test | t(20) = 0.15,  p = 0.881, d = 0.06 |  |  |
|  |  |  |  |  | Chd2^+/cKO^ | | 20.50 | 1.53 |  |  |  |  |
| S3H |  | Control = 12 Chd2^+/cKO^ = 10 | % time in center zone (s) | | Control | | 14.66 | 2.39 | Two-tailed  t-test | t(20) = 0.1192,  p = 0.907, d = 0.05 |  |  |
|  |  |  |  |  | Chd2^+/cKO^ | | 14.33 | 1.11 |  |  |  |  |
| S3I | OFT female | Control = 6 Chd2^+/cKO^ = 6 | Distance | | Control | | 28.43 | 3.44 | Two-tailed  t-test | t(10) = 0.048,  p = 0.963, d = 0.03 |  |  |
|  |  |  |  |  | Chd2^+/cKO^ | | 28.21 | 2.95 |  |  |  |  |
| S3J |  | Control = 6 Chd2^+/cKO^ = 6 | % time in center zone (s) | | Control | | 14.35 | 1.51 | Two-tailed  t-test | t(10) = 0.16,  p = 0.878, d = 0.092 |  |  |
|  |  |  |  |  | Chd2^+/cKO^ | | 13.79 | 3.25 |  |  |  |  |
| S3L | EPM male | WT = 9 Chd2^+/-^ = 13 | Open arm | Time (s) | WT | | 106.04 | 13.60 | Two-tailed  t-test | t(20) = 0.5812,  p = 0.5676, d = 0.24 |  |  |
|  |  |  |  |  | Chd2^+/-^ | | 116.17 | 11.03 |  |  |  |  |
| S3M |  | WT = 9 Chd2^+/-^ = 13 | Closed arm | Time (s) | WT | | 430.31 | 24.97 | Two-tailed  t-test | t(20) =0.5775,  p = 0.5701, d = 0.25 |  |  |
|  |  |  |  |  | Chd2^+/-^ | | 412.58 | 18.84 |  |  |  |  |
| S3N | EPM female | WT = 12 Chd2^+/-^ = 10 | Open arm | Time (s) | WT | | 89.69 | 8.86 | Two-tailed  t-test | t(20) = 1.48,  p = 0.1554, d = 0.63 |  |  |
|  |  |  |  |  | Chd2^+/-^ | | 107.57 | 7.91 |  |  |  |  |
| S3O |  | WT = 12 Chd2^+/-^ = 10 | Closed arm | Time (s) | WT | | 462.73 | 12.34 | Two-tailed  t-test | t(20) = 1.219,  p = 0.2370, d = 0.52 |  |  |
|  |  |  |  |  | Chd2^+/-^ | | 442.16 | 11.04 |  |  |  |  |
| S3Q | EPM male | Control = 12 Chd2^+/cKO^ = 10 | Open arm | Time (s) | Control | | 112.92 | 15.76 | Two-tailed  t-test | t(20) = 1.23,  p = 0.235, d = 0.52 |  |  |
|  |  |  |  |  | Chd2^+/cKO^ | | 140.46 | 15.74 |  |  |  |  |
| S3R |  | Control = 12 Chd2^+/cKO^ = 10 | Closed arm | Time (s) | Control | | 406.88 | 22.08 | Two-tailed  t-test | t(20) = 1.008,  p = 0.3256, d = 0.43 |  |  |
|  |  |  |  |  | Chd2^+/cKO^ | | 377.83 | 17.08 |  |  |  |  |
| S3S | EPM female | Control = 6 Chd2^+/cKO^ = 6 | Open arm | Time (s) | Control | | 81.77 | 10.05 | Two-tailed  t-test | t(10) = 1.69,  p = 0.121, d = 0.98 |  |  |
|  |  |  |  |  | Chd2^+/cKO^ | | 107.48 | 11.38 |  |  |  |  |
| S3T |  | Control = 6 Chd2^+/cKO^ = 6 | Closed arm | Time (s) | Control | | 465.38 | 19.49 | Two-tailed  t-test | t(10) = 3.404,  p = 0.0673, d = 1.96 |  |  |
|  |  |  |  |  | Chd2^+/cKO^ | | 387.92 | 11.75 |  |  |  |  |
| S3V | Marble burying male | WT = 9 Chd2^+/-^ = 13 | Buried marbles | | WT | | 15.11 | 0.75 | Two-tailed  t-test | t(20) = 1.951,  p = 0.0652, d = 0.83 |  |  |
|  |  |  |  |  | Chd2^+/-^ | | 12.31 | 1.07 |  |  |  |  |
| S3W |  | Control = 6 Chd2^+/cKO^ = 6 | Buried marbles | | Control | | 14.75 | 1.03 | Two-tailed  t-test | t(10) = 0.469,  p = 0.6495, d = 0.27 |  |  |
|  |  |  |  |  | Chd2^+/cKO^ | | 15.75 | 0.75 |  |  |  |  |
| S3X | Marble burying female | WT = 12 Chd2^+/-^ = 10 | Buried marbles | | WT | | 13.25 | 0.60 | Two-tailed  t-test | t(20) = 1.610,  p = 0.1232, d = 0.69 |  |  |
|  |  |  |  |  | Chd2^+/-^ | | 11.60 | 0.86 |  |  |  |  |
| S3Y |  | Control = 6 Chd2^+/cKO^ = 6 | Buried marbles | | Control | | 12.00 | 1.00 | Two-tailed  t-test | t(10) = 0.55, p = 0.592, d = 0.32 |  |  |
|  |  |  |  |  | Chd2^+/cKO^ | | 11.00 | 1.51 |  |  |  |  |
| S3Z | FST male | WT = 9 Chd2^+/-^ = 13 | Time immobile (s) | | WT | | 41.11 | 4.34 | Two-tailed  t-test | t(20) = 0.37,  p = 0.7186, d = 0.16 |  |  |
|  |  |  |  |  | Chd2^+/-^ | | 43.92 | 5.63 |  |  |  |  |
| S3AA |  | Control = 12 Chd2^+/cKO^ = 10 | Time immobile (s) | | Control | | 46.00 | 11.71 | Two-tailed  t-test | t(20) = 0.298,  p = 0.769, d = 0.13 |  |  |
|  |  |  |  |  | Chd2^+/cKO^ | | 41.30 | 10.00 |  |  |  |  |
| S3BB | FST female | WT = 12 Chd2^+/-^ = 10 | Time immobile (s) | | WT | | 43.50 | 8.34 | Two-tailed  t-test | t(20) = 0.1896,  p = 0.8515, d = 0.081 |  |  |
|  |  |  |  |  | Chd2^+/-^ | | 41.70 | 8.34 |  |  |  |  |
| S3CC |  | Control = 6 Chd2^+/cKO^ = 6 | Time immobile (s) | | Control | | 32.83 | 9.70 | Two-tailed  t-test | t(10) = 0.54,  p = 0.602, d = 0.31 |  |  |
|  |  |  |  |  | Chd2^+/cKO^ | | 38.33 | 3.17 |  |  |  |  |
| S6A | Sociability male | Vehicle  WT = 8 Chd2^+/-^ = 6  L-838,417  WT = 8 Chd2^+/-^ = 6 | Total obs.  time (s) | Vehicle | WT | | 218.13 | 8.92 | Two-way  RM ANOVA | Genotype, F (1, 12) = 0.1053, p = 0.75, η²_p_ = 0.0065 Treatment, F (1, 12) = 0.001040, p = 0.97,  η²_p_ = 8.66E-05 Interaction, F (1, 12) = 0.08135, p = 0.78, η²_p_ = 0.0067 | Tukey | Vehicle, WT vs Chd2^+/-^, p = 0.99 L-838,417, WT vs Chd2^+/-^, p = 0.67 |
|  |  |  |  |  | Chd2^+/-^ | | 218.17 | 4.63 |  |  |  |  |
|  |  |  |  | L-838,417 | WT | | 220.25 | 7.71 |  |  |  |  |
|  |  |  |  |  | Chd2^+/-^ | | 215.50 | 7.55 |  |  |  |  |
| S6B |  | Vehicle  WT = 8 Chd2^+/-^ = 6  L-838,417  WT = 8 Chd2^+/-^ = 6 | Distance | Vehicle | WT | | 2.45 | 0.10 | Two-way  RM ANOVA | Genotype, F (1, 12) = 0.2981, p = 0.6, η²_p_ = 0.0095 Treatment, F (1, 12) = 0.1911, p = 0.67, η²_p_ = 0.016 Interaction, F (1, 12) = 0.6285, p = 0.44, η²_p_ = 0.05 | Tukey | Vehicle, WT vs Chd2^+/-^, p = 0.35 L-838,417, WT vs Chd2^+/-^, p = 0.7 |
|  |  |  |  |  | Chd2^+/-^ | | 2.59 | 0.11 |  |  |  |  |
|  |  |  |  | L-838,417 | WT | | 2.50 | 0.11 |  |  |  |  |
|  |  |  |  |  | Chd2^+/-^ | | 2.44 | 0.08 |  |  |  |  |
| S6C | Sociability female | Vehicle  WT = 6 Chd2^+/-^ = 7  L-838,417  WT = 6 Chd2^+/-^ = 7 | Total obs.  time (s) | Vehicle | WT | | 219.33 | 6.59 | Two-way  RM ANOVA | Genotype, F (1, 11) = 0.2353, p = 0.64, η²_p_ = 0.020 Treatment, F (1, 11) = 0.02803, p = 0.87, η²_p_ = 0.0025 Interaction, F (1, 11) = 0.1048, p = 0.75, η²_p_ = 0.0094 | Tukey | Vehicle, WT vs Chd2^+/-^, p = 0.57 L-838,417, WT vs Chd2^+/-^, p = 0.92 |
|  |  |  |  |  | Chd2^+/-^ | | 224.43 | 7.40 |  |  |  |  |
|  |  |  |  | L-838,417 | WT | | 220.43 | 2.42 |  |  |  |  |
|  |  |  |  |  | Chd2^+/-^ | | 221.33 | 2.63 |  |  |  |  |
| S6D |  | Vehicle  WT = 6, Chd2^+/-^ = 7  L-838,417  WT = 6  Chd2^+/-^ = 7 | Distance | Vehicle | WT | | 2.50 | 0.08 | Two-way  RM ANOVA | Genotype, F (1, 11) = 3.014, p = 0.11, η²_p_ = 0.17 Treatment, F (1, 11) = 0.0006678, p = 0.98,  η²_p_ = 6.07E-05 Interaction, F (1, 11) = 0.8389, p = 0.38, η²_p_ = 0.071 | Tukey | Vehicle, WT vs Chd2^+/-^, p = 0.65 L-838,417, WT vs Chd2^+/-^, p = 0.08 |
|  |  |  |  |  | Chd2^+/-^ | | 2.55 | 0.07 |  |  |  |  |
|  |  |  |  | L-838,417 | WT | | 4.76 | 0.10 |  |  |  |  |
|  |  |  |  |  | Chd2^+/-^ | | 5.75 | 0.08 |  |  |  |  |

**References**

1. Wang Z, Mu W, Zhong J, Xu R, Liu Y, Zhao G, et al. (2024) Vascular smooth muscle cell PRDM16 regulates circadian variation in blood pressure. J Clin Invest. 135:e183409.
2. Evdokimovskii EV, Jeon R, Park S, Pimenov OY, Alekseev AE (2021) Role of α2-Adrenoceptor Subtypes in Suppression of L-Type Ca2+ Current in Mouse Cardiac Myocytes. Int J Mol Sci. 22:4135.
3. Liu D, Liang M, Zhu L, Zhou TT, Wang Y, Wang R, et al. (2021) Potential Ago2/miR-3068-5p Cascades in the Nucleus Accumbens Contribute to Methamphetamine-Induced Locomotor Sensitization of Mice. Front Pharmacol. 12:708034.
4. Colombo E, Galli R, Cossu G, Gécz J, Broccoli V. (2004) Mouse orthologue of ARX, a gene mutated in several X-linked forms of mental retardation and epilepsy, is a marker of adult neural stem cells and forebrain GABAergic neurons. Dev Dyn. 231(3):631-9.
5. Parag RR, Yamamoto T, Saito K, Zhu D, Yang L, Van Meir EG (2025) Novel Isoforms of Adhesion G Protein-Coupled Receptor B1 (ADGRB1/BAI1) Generated from an Alternative Promoter in Intron 17. Mol Neurobiol. 62(1):900-917.
6. Sardar D, Chen HC, Reyes A, Varadharajan S, Jain A, Mohila C, et al. (2022) Sox9 directs divergent epigenomic states in brain tumor subtypes. Proc Natl Acad Sci U S A. 119(29):e2202015119.
7. Peng Z, Heath J, Drachenberg C, Raufman JP, Xie G (2013) Cholinergic muscarinic receptor activation augments murine intestinal epithelial cell proliferation and tumorigenesis. BMC Cancer. 13:204.
8. Zhang J, Chen SR, Chen H, Pan HL (2018) RE1-silencing transcription factor controls the acute-to-chronic neuropathic pain transition and Chrm2 receptor gene expression in primary sensory neurons. J Biol Chem. 293:19078-19091.
9. Niwa Y, Kanda GN, Yamada RG, Shi S, Sunagawa GA, Ukai-Tadenuma M, et al. (2018) Muscarinic Acetylcholine Receptors Chrm1 and Chrm3 Are Essential for REM Sleep. Cell Rep. 13:1402.
10. Smith LIF, Zhao Z, Walker J, Lightman S, Spiga F. (2021) Activation and expression of endogenous CREB-regulated transcription coactivators (CRTC) 1, 2 and 3 in the rat adrenal gland. J Neuroendocrinol. 33(1):e12920.
11. Pratt T, Davey JW, Nowakowski TJ, Raasumaa C, Rawlik K, McBride D, et al. (2012) The expression and activity of β-catenin in the thalamus and its projections to the cerebral cortex in the mouse embryo. BMC Neurosci.13:20.
12. Amar M, Pramod AB, Yu NK, Herrera VM, Qiu LR, Moran-Losada P, et al. (2021) Autism-linked Cullin3 germline haploinsufficiency impacts cytoskeletal dynamics and cortical neurogenesis through RhoA signaling. Mol Psychiatry. 26(7):3586-3613.
13. Li Q, Zhang P, Zhang C, Wang Y, Wan R, Yang Y, et al. (2014) DDX3X regulates cell survival and cell cycle during mouse early embryonic development. J Biomed Res. 28(4):282-91.
14. Gupta P, Uner OE, Nayak S, Grant GR, Kalb RG (2018) SAP97 regulates behavior and expression of schizophrenia risk enriched gene sets in mouse hippocampus. PLoS One. 13(7):e0200477.
15. Ferland RJ, Cherry TJ, Preware PO, Morrisey EE, Walsh CA. (2003) Characterization of Foxp2 and Foxp1 mRNA and protein in the developing and mature brain. J Comp Neurol. 460(2):266-79.
16. Yeung RK, Xiang ZH, Tsang SY, Li R, Ho TYC, Li Q, et al. (2018) Gabrb2-knockout mice displayed schizophrenia-like and comorbid phenotypes with interneuron–astrocyte–microglia dysregulation. Transl Psychiatry. 8:128.
17. Bosman LW, Heinen K, Spijker S, Brussaard AB (2005) Mice lacking the major adult GABAA receptor subtype have normal number of synapses, but retain juvenile IPSC kinetics until adulthood. J Neurophysiol. 94:338-46.
18. Sailaja BS, Cohen-Carmon D, Zimmerman G, Soreq H, Meshorer E (2012) Stress-induced epigenetic transcriptional memory of acetylcholinesterase by HDAC4. Proc Natl Acad Sci U S A. 26;109(52).
19. Fei X, Dou YN, Wang L, Wu X, Huan Y, Wu S, et al. (2022) Homer1 promotes the conversion of A1 astrocytes to A2 astrocytes and improves the recovery of transgenic mice after intracerebral hemorrhage. J Neuroinflammation. 19(1):67.
20. Wang CC, Man GC, Chu CY, Borchert A, Ugun-Klusek A, et al.(2014) Serotonin Receptor 6 Mediates Defective Brain Development in Monoamine Oxidase A-deficient Mouse Embryos. J Biol Chem. 289:8252-63.
21. Samanta MK, Gayen S, Harris C, Maclary E, Murata-Nakamura Y, Malcore RM, et al. (2022) Activation of Xist by an evolutionarily conserved function of KDM5C demethylase. Nat Commun. 13(1):2602.
22. Nakamura T, Yoshihara T, Tanegashima C, Kadota M, Kobayashi Y, Honda K, et al. (2024) Transcriptomic dysregulation and autistic-like behaviors in Kmt2c haploinsufficient mice rescued by an LSD1 inhibitor. Mol Psychiatry. 29(9):2888-2904.
23. Ahmed O, Ekumi KM, Nardi FV, Maisumu G, Moussawi K, Lazartigues ED, et al. (2024) Stable, neuron-specific gene expression in the mouse brain. J Biol Eng. 18:8.
24. Kim J, Chun Y, Ramirez CB, Hoffner LA, Jung S, Jang KH, et al. (2023) MAPK13 stabilization via m^6^A mRNA modification limits anticancer efficacy of rapamycin. J Biol Chem. 299(9):105175.
25. Wang X, Wei H, Hu Z, Jiang J, Dong X, Zhu J, et al. (2025) Chronic stress induces depression through MDGA1-Neuroligin2 mediated suppression of inhibitory synapses in the lateral habenula. Theranostics. 15(5):1842-1863.
26. Haque F, Honjo T, Begum NA. (2022) XLID syndrome gene Med12 promotes Ig isotype switching through chromatin modification and enhancer RNA regulation. Sci Adv.8(47):eadd1466.
27. Runkel F, Rohlmann A, Reissner C, Brand SM, Missler M. (2013) Promoter-like sequences regulating transcriptional activity in neurexin and neuroligin genes. J Neurochem. 127(1):36-47.
28. Haile MT, Khoja S, de Carvalho G, Hunt RF, Chen LY. Conditional deletion of Neurexin-2 alters neuronal network activity in hippocampal circuitries and leads to spontaneous seizures. (2023) Transl Psychiatry. 13(1):97.
29. Pisansky MT, Hanson LR, Gottesman II, Gewirtz JC (2017) Oxytocin enhances observational fear in mice. Nat Commun. 8:2102.
30. de Sena Cortabitarte A, Berkel S, Cristian FB, Fischer C, Rappold GA. (2018) A direct regulatory link between microRNA-137 and SHANK2: implications for neuropsychiatric disorders. J Neurodev Disord. 10(1):15.
31. Nestor CC, Qiu J, Padilla SL, Zhang C, Bosch MA, Fan W, et al. (2016) Optogenetic Stimulation of Arcuate Nucleus Kiss1 Neurons Reveals a Steroid-Dependent Glutamatergic Input to POMC and AgRP Neurons in Male Mice. Mol Endocrinol. 30:630-44.
32. Li F, Liang J, Tang D. (2018) Brahma-related gene 1 ameliorates the neuronal apoptosis and oxidative stress induced by oxygen-glucose deprivation/reoxygenation through activation of Nrf2/HO-1 signaling. Biomed Pharmacother. 108:1216-1224.
33. Kawaai K, Ando H, Satoh N, Yamada H, Ogawa N, Hirose M, et al. (2017) Splicing variation of Long-IRBIT determines the target selectivity of IRBIT family proteins. Proc Natl Acad Sci U S A. 114(15):3921-3926.
34. Anderson JS, Lodigiani AL, Barbaduomo CM, Beegle JR. (2024) Hematopoietic stem cell gene therapy for the treatment of SYNGAP1-related non-specific intellectual disability. J Gene Med. 26(7):e3717.
35. Herron RS, Kunisky AK, Madden JR, Anyaeche VI, Maung MZ, Hwang HW. (2023) A twin UGUA motif directs the balance between gene isoforms through CFIm and the mTORC1 signaling pathway. Elife. 12:e85036.
36. Lee D, Chen W, Kaku HN, Zhuo X, Chao ES, Soriano A, et al. (2023) Antisense oligonucleotide therapy rescues disturbed brain rhythms and sleep in juvenile and adult mouse models of Angelman syndrome. Elife. 12:e81892.
